# NOD2-mediated Suppression of CD55 on Neutrophils Enhances C5a Generation During Polymicrobial Sepsis

**DOI:** 10.1371/journal.ppat.1003351

**Published:** 2013-05-09

**Authors:** Sae Jin Oh, Ji Hyung Kim, Doo Hyun Chung

**Affiliations:** 1 Department of Pathology, Seoul National University College of Medicine, Seoul, Korea; 2 Laboratory of Immune Regulation in Department of Biomedical Sciences, Seoul National University College of Medicine, Seoul, Korea; University of São Paulo, Brazil

## Abstract

Nucleotide-binding oligomerization domain (NOD) 2 is a cytosolic protein that plays a defensive role in bacterial infection by sensing peptidoglycans. C5a, which has harmful effects in sepsis, interacts with innate proteins. However, whether NOD2 regulates C5a generation during sepsis remains to be determined. To address this issue, cecal ligation & puncture (CLP)-induced sepsis was compared in wild type and Nod2^−/−^ mice. Nod2^−/−^ mice showed lower levels of C5a, IL-10, and IL-1β in serum and peritoneum, but higher survival rate during CLP-induced sepsis compared to wild type mice. Injection of recombinant C5a decreased survival rates of Nod2^−/−^ mice rate during sepsis, whereas it did not alter those in wild type mice. These findings suggest a novel provocative role for NOD2 in sepsis, in contrast to its protective role during bacterial infection. Furthermore, we found that NOD2-mediated IL-10 production by neutrophils enhanced C5a generation by suppressing CD55 expression on neutrophils in IL-1β-dependent and/or IL-1β-independent manners, thereby aggravating CLP-induced sepsis. SB203580, a receptor-interacting protein 2 (RIP2) inhibitor downstream of NOD2, reduced C5a generation by enhancing CD55 expression on neutrophils, resulting in attenuation of polymicrobial sepsis. Therefore, we propose a novel NOD2-mediated complement cascade regulatory pathway in sepsis, which may be a useful therapeutic target.

## Author Summary

Nucleotide-binding oligomerization domain (NOD) 2 is a cytosolic protein that senses peptidoglycans of bacteria and exerts a defensive effect on bacterial infection. Sepsis is a complex dysregulated inflammatory response in bacterial infection, causing multiple organ dysfunction, coagulopathy, and fatal outcome. C5a, which has harmful effects in sepsis, interacts with innate proteins. However, it remains unclear the mechanism by which NOD2 affects sepsis responses *in vivo* by regulating C5a generation. Here, we demonstrate that NOD2 enhances C5a generation by IL-10-mediated suppression of CD55 expression on neutrophils, thereby aggravating polymicrobial sepsis. These findings suggest a provocative role for NOD2 in sepsis, in contrast to its protective role during bacterial infection. Therefore, we propose that a novel NOD2-mediated complement cascade regulatory pathway in neutrophils may be a useful therapeutic target for sepsis.

## Introduction

Sepsis is a complex dysregulated inflammatory response in infection, which causes multiple organ dysfunction and coagulopathy, often resulting in death [1], [2]. Pro-inflammatory cytokines contribute to an overwhelming inflammatory immune response in the early phase of sepsis, whereas anti-inflammatory cytokines are involved in the late-phase immune response [1], [3]. Studies have shown that C5a, a complement protein, has harmful effects during sepsis, although the complement is crucial for clearance of infectious agents [4]–[8]. During sepsis, C5a causes multiple organ failure, cardiomyopathy, and imbalanced coagulation [9]. Therefore, C5a is generally accepted as a crucial target for therapeutic approaches in sepsis. Nevertheless, the mechanisms by which C5a is regulated in sepsis remain unclear.

Nod-like receptors and toll-like receptors (TLRs) are a family of innate proteins that trigger innate immune activation by recognizing pathogen-associated molecular patterns [10]. NOD2 genetic mutations in humans have been implicated in Crohn's disease, Blau syndrome, sarcoidosis, and graft-versus-host disease [11]–[14]. With respect to bacterial infection, NOD2-mediated peptidoglycan sensing regulates mononuclear cell recruitment and chemokine production, which promotes clearance of Streptococcus pneumonia [15]. Moreover, NOD2 activates the autophagy process, thereby confining intracellular bacteria within intracellular autophagosomes and subsequently restricting the infection [16], [17]. Consistent with these data, Nod2-deficient mice are susceptible to Listeria monocytogenes, indicating that NOD2 plays a defensive role during bacterial infection [18]. Moreover, very recent study suggests that polymorphisms in the NOD2/Card15 gene might be related with susceptibility to sepsis in children [19]. However, it remains unclear whether NOD2 play crucial role in the pathogenesis of sepsis. It has been demonstrated that various TLR ligands regulate cytokine production in a complement-dependent manner, suggesting that crosstalk between innate proteins and the complement system makes a crucial contribution to immune response regulation *in vivo* [20]. Thus, we hypothesize that NOD2 regulates C5a generation via crosstalk with the complement system during sepsis. To address this hypothesis, we investigated whether NOD2 regulates C5a generation during sepsis. Our results indicate that NOD2-mediated signals enhance C5a generation by suppressing CD55 expression on neutrophils through IL-1β-dependent or IL-1β-independent IL-10 production during polymicrobial sepsis.

## Results

### NOD2-mediated signals enhance C5a generation, thereby promoting sepsis

**Figure 1:**
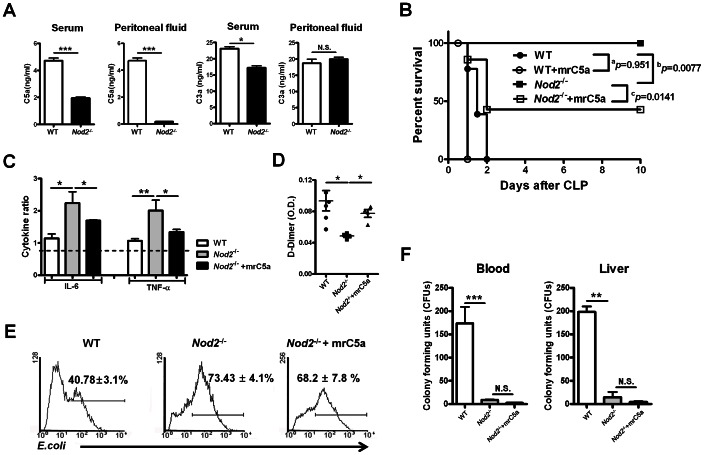
Nucleotide-binding oligomerization domain (Nod2)^−/−^ mice exhibit attenuated cecal ligation and puncture (CLP)-induced sepsis through suppression of C5a generation. To induce sepsis, the cecum of wild-type (WT) or Nod2^−/−^ mice was ligated and punctured. (A) Serum and peritoneal C5a and C3a levels were evaluated in WT and Nod2^−/−^ mice 24 h after CLP (n = 4). (B) The percentages of surviving mice were estimated during CLP-induced sepsis (^a^P = 0.951[not significant], ^b^P = 0.0077, ^c^P = 0.0141, log-rank test; WT [n = 12], WT mice injected with recombinant C5a [n = 8], Nod2^−/−^ [n = 8], Nod2^−/−^ mice injected with recombinant C5a [ n = 8]) (C) Peritoneal cells obtained from WT (n = 4), Nod2^−/−^ (n = 4), or Nod2^−/−^ mice injected with recombinant C5a (n = 6) 4 and 12 h after CLP were incubated with LPS or PBS for 6 h and cytokine levels were measured. The responsiveness of peritoneal cells to LPS was determined by estimating the ratios of individual cytokines produced in response to LPS versus PBS. (D) Serum D-dimer levels were measured in WT (n = 5), Nod2^−/−^(n = 5), Nod2^−/−^ mice injected with recombinant C5a (n = 4) 24 h after CLP. (E) Phagocytosis activity of peritoneal cells obtained from mice 24 h after CLP was determined by measuring the percentage of cells with intracellular FITC-conjugated *E. coli* after 15 min incubation. (n = 3) (F) Bacterial CFUs were counted using blood and liver homogenates obtained from mice 24 h after CLP. (n = 3) *P<0.05, **P<0.01, ***P<0.001 (two-tailed unpaired t-test [a, c–f]) for WT B6 vs. Nod2^−/−^ mice and Nod2^−/−^ vs. Nod2^−/−^ mice injected with recombinant C5a. Results shown are representative of three independent experiments except for (B) (mean and SEM).

To investigate whether NOD2 regulates C5a generation during sepsis, we performed CLP in wild-type (WT) and Nod2^−/−^ mice. Serum and peritoneal C5a levels were lower in Nod2^−/−^ than in WT mice during sepsis, whereas C3a levels were similar (Fig. 1A). All Nod2^−/−^ mice were alive up to 10 days after CLP, whereas all WT mice died within 2 days. However, ELISA system for C5a might detect C5 in some cases, although cross reactivity for C5a and C5 in detection system does not usually occur. Thus, to confirm harmful effects of NOD2-mediated C5a generation on sepsis, we injected WT and Nod2^−/−^ mice with recombinant (r)C5a during CLP-induced sepsis. Injection of Nod2^−/−^ mice with rC5a decreased survival rates during sepsis, whereas rC5a had no effect on survival of WT mice (Fig. 1B). These findings suggest that NOD2-mediated C5a generation contributes to sepsis development and severity. NOD2 was recently reported to affect composition of the host microbiota in mice and humans [21]. To rule out differences in the cecal bacterial composition of WT and Nod2^−/−^ mice might affect sepsis, cecal contents obtained from WT or Nod2^−/−^ mice were injected i.p. into WT and Nod2^−/−^ mice after their ceca had been ligated but not punctured and WT and Nod2^−/−^ mice were cohoused for 4 weeks. The survival rates after injection of Nod2^−/−^ or WT cecal contents were lower in WT mice than Nod2^−/−^ mice (Fig. S1). Moreover, survival rates of cohoused WT mice were lower than cohoused Nod2^−/−^ mice during CLP-induced sepsis (Fig. S1), indicating that difference in intestinal microbiota between two mouse groups minimally contributes to septic responses because gut microbiota of cohoused mice are replaced by each other [22]. Collectively, these findings suggest that NOD2-mediated signals enhance generation of C5a, but not C3a, thereby enhancing the systemic inflammatory response.

To determine the effect of C5a on immune responses in WT and Nod2^−/−^ mice during sepsis, we investigated neutrophil dysfunction by measuring the responsiveness of immune cells to LPS, phagocytic activity, CFUs, and serum D-dimer levels after CLP. Total peritoneal cells obtained from Nod2^−/−^ mice 24 h after CLP produced higher IL-6 and TNF-α levels to LPS than did WT peritoneal cells (Fig. 1C). Moreover, serum D-dimer levels in Nod2^−/−^ mice were lower than those in WT mice (Fig. 1D). However, total peritoneal cells obtained from Nod2^−/−^ mice engulfed more FITC-conjugated *Escherichia coli* than did WT peritoneal cells (Fig. 1E). Consistent with these findings, culturable bacterial CFU levels in blood and liver homogenates were higher in WT than in Nod2^−/−^ mice (Fig. 1F). rC5a administration to Nod2^−/−^ mice with CLP reversed the response to LPS by peritoneal cells and serum D-dimer levels, but not phagocytosis activity or bacterial CFUs. These findings suggest that NOD2-mediated signals trigger immune cell dysfunction and coagulopathy by enhancing C5a levels during sepsis, whereas the NOD2-mediated immune response regulates bacterial phagocytic activity and CFU levels in a C5a-independent manner.

### NOD2-mediated signals induce IL-1β and IL-10 production by neutrophils during sepsis

**Figure 2:**
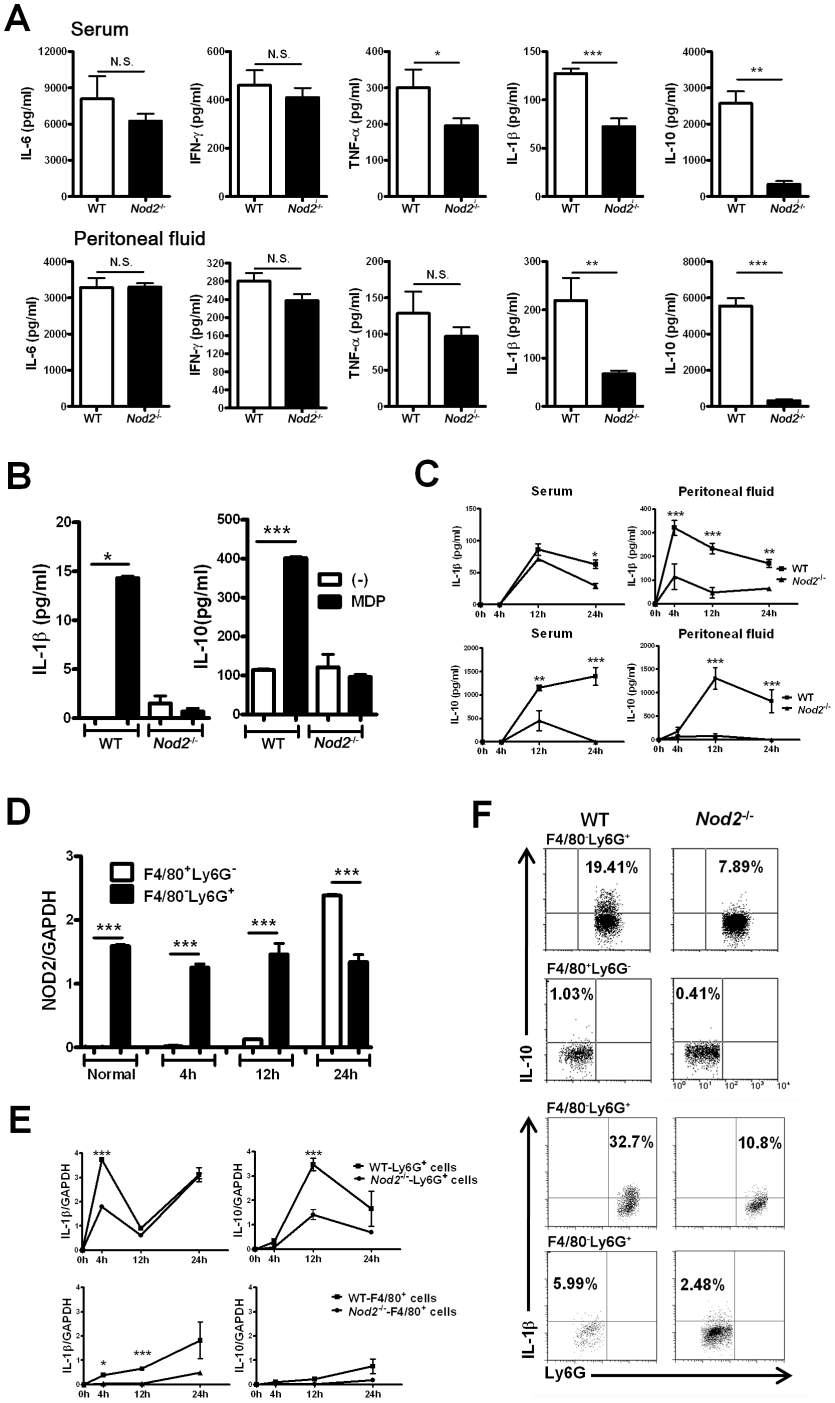
Nucleotide-binding oligomerization domain (NOD)2-mediated signals induce IL-1β and IL-10 production by peritoneal neutrophils during cecal ligation and puncture (CLP)-induced sepsis. (A) Serum and peritoneal IL-6, IFN-γ, TNF-α, IL-1β, and IL-10 levels were measured in WT (n = 4) and Nod2^−/−^ (n = 4) mice 24 h after CLP. (B) Peritoneal cells were obtained from WT or Nod2^−/−^ mice 4–6 h after injection with thioglycollate and incubated with or without MDP for 24 h. The IL-1β and IL-10 concentrations in culture supernatants were measured. (C) Serum and peritoneal IL-1β and IL-10 levels were estimated in WT (n = 4) and Nod2^−/−^ (n = 4) mice 4, 12, and 24 h after CLP using ELISA. (D) The NOD2 expression pattern was estimated in sorted F4/80^−^Ly-6G^+^ and F4/80^+^Ly-6G^−^ peritoneal cells of WT mice 4, 12, and 24 h after CLP by real-time PCR. (E) IL-1β and IL-10 transcript levels were evaluated in sorted F4/80^−^Ly-6G^+^ and F4/80^+^Ly-6G^−^ peritoneal cells from WT and Nod2^−/−^ mice 4, 12, and 24 h after CLP. (F) Intracellular IL-1β and IL-10 expression in gated F4/80^−^Ly-6G^+^ and F4/80^+^Ly-6G^−^ peritoneal cells was analyzed by flow cytometry 2, and 12 h after CLP, respectively. (n = 3) *P<0.05, **P<0.01, ***P<0.001 for WT vs. Nod2^−/−^ mice (two-tailed unpaired t-test [a, b], two-way ANOVA [c, e]) Results shown are representative of three independent experiments (mean and SEM).

To explore the mechanism by which NOD2 enhances C5 generation during sepsis, the serum and peritoneal levels of various cytokines in WT and Nod2^−/−^ mice were estimated after CLP. The serum and peritoneal IL-1β and IL-10 levels of WT mice were significantly higher than those of Nod2^−/−^ mice, whereas IL-6 and IFN-γ levels in WT mice were similar to those in Nod2^−/−^ mice (Fig. 2A). Serum TNF-α levels were higher in WT than Nod2^−/−^ mice, whereas its peritoneal levels were similar in two mouse groups. To estimate IL-1β and IL-10 production by peritoneal cells, we obtained total peritoneal cells from WT and Nod2^−/−^ mice 4–6 h after injection with thioglycollate. Upon MDP, an NOD2 agonist treatment, WT peritoneal cells produced IL-1β and IL-10, whereas NOD2-deficient cells produced minimal IL-1β and IL-10 (Fig. 2B). A kinetic analysis revealed that IL-1β and IL-10 levels in peritoneal fluid peaked 4 and 12 h after CLP, respectively, and then decreased gradually (Fig. 2C). Serum IL-1β levels peaked 12 h after CLP and were significantly higher in WT than Nod2^−/−^ mice at 24 h, whereas serum IL-10 levels in WT mice increased continuously from 4 to 24 h after CLP, and were significantly higher than those in Nod2^−/−^ mice. Subset analysis revealed that F4/80^−^Ly-6G^+^ neutrophils and F4/80^+^Ly-6G^−^ macrophages were major cells infiltrated into peritoneum during sepsis (Fig. S2) and the numbers of these cells was similar in Nod2^−/−^ and WT mice (data not shown). The percentages of neutrophils peaked and macrophages showed the lowest percentages in Nod2^−/−^ and WT mice 4 h after CLP, and the percentages of these cells were similar in WT and Nod2^−/−^ mice before, 4, 12, and 24 h after CLP (Fig. S2). Based on these findings, NOD2 expression was investigated in sorted F4/80^+^Ly-6G^−^ macrophages and F4/80^−^Ly-6G^+^ neutrophils from peritoneum of WT mice with CLP (Fig. 2D). Real-time PCR analysis revealed that NOD2 expression in F4/80^−^Ly-6G^+^ neutrophils was constitutive and sustained during sepsis, whereas F4/80^+^Ly-6G^−^ macrophages showed low expression levels of NOD2 before, and 4 and 12 h after CLP, but highly expressed NOD2 24 h after CLP, which was consistent with expression pattern of NOD2 in blotting assay (S3A and B). These findings indicate that F4/80^−^Ly-6G^+^ neutrophils rather than F4/80^+^Ly-6G^−^ macrophages in the peritoneum predominantly express NOD2 during early and intermediate stages of sepsis. Consistent with the kinetics of the IL-1β and IL-10 levels, peritoneal F4/80^−^Ly-6G^+^ neutrophils from WT mice showed high IL-1β mRNA levels at 4 and 24 h, but low transcriptional levels at 12 h (Fig. 2E). In contrast, peritoneal F4/80^+^Ly-6G^−^ macrophages produced high levels of IL-1β at 24 h. Unlike IL-1β, peritoneal F4/80^−^Ly-6G^+^ neutrophils from WT mice predominantly produced IL-10 at 12 h. Although the kinetics of IL-1β and IL-10 production were similar in Nod2^−/−^ and WT mice, individual cytokine levels were much lower in the former, which were consistent with results in intracellular staining (Fig. 2F). To investigate whether peritoneal neutrophils produce IL-1β and IL-10 during sepsis, peritoneal F4/80^−^Ly-6G^+^ neutrophils and F4/80^+^Ly-6G^−^ macrophages from Nod2^−/−^ and WT mice with CLP were obtained, sorted, and cultured for 24 h without stimulation. F4/80^−^Ly-6G^+^ neutrophils from WT mice produced larger amount of IL-1β and IL-10 than WT F4/80^+^Ly-6G^−^ macrophages did (Fig. S4A). In contrast, F4/80^−^Ly-6G^+^ neutrophils and F4/80^+^Ly-6G^−^ macrophages from Nod2^−/−^ mice minimally produced IL-1β and IL-10. Furthermore, neutrophil depletion using anti-Ly-6G mAb in WT mice reduced the levels of IL-1β and IL-10 in serum and peritoneum, which was dependent on depletion time points (Fig. S4B and C). Combined *in vitro* and depletion experiments, it is suggested that peritoneal neutrophils rather than macrophages directly produce IL-1β and IL-10 at different time points during sepsis, although neutrophils might interact with macrophages or monocytes to produce various cytokines during CLP-induced sepsis.

### NOD2-mediated IL-1β production by neutrophils enhances C5a generation during sepsis in an IL-10-dependent manner

**Figure 3:**
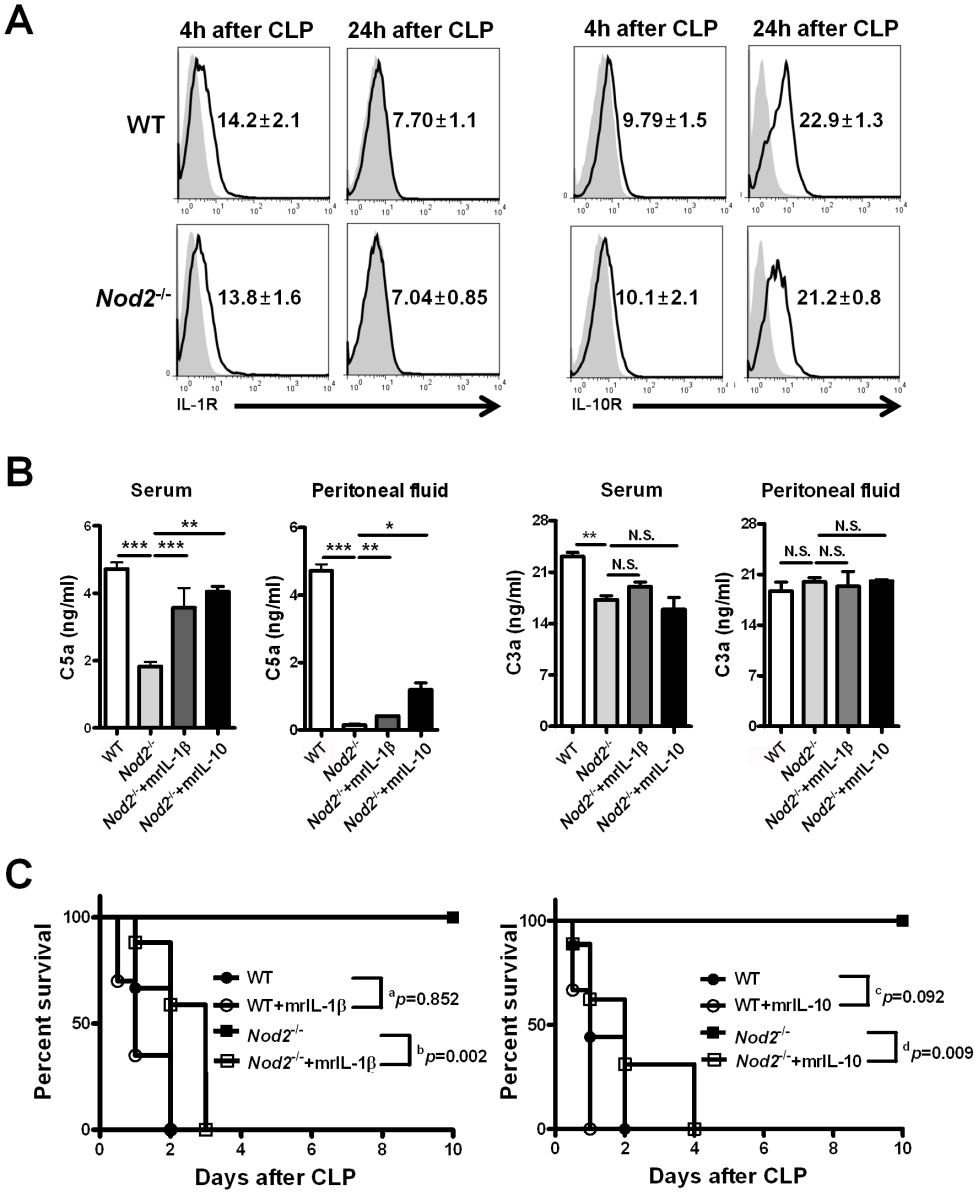
IL-1β-dependent IL-10 production mediated by nucleotide-binding oligomerization domain (NOD) 2 enhances C5a generation during cecal ligation and puncture (CLP)-induced sepsis. (A) IL-1 and IL-10 receptor expression was estimated on total peritoneal cells in terms of mean fluorescence intensity (MFI) from WT (n = 3) and Nod2^−/−^ (n = 3) mice 4 h and 24 h after CLP. (B) Serum and peritoneal C5a and C3a levels were measured 24 h after CLP in WT (n = 4) and Nod2^−/−^ (n = 4) mice injected with recombinant IL-1β or IL-10 prior to CLP. (C) The survival percentages of WT and Nod2^−/−^mice injected with recombinant IL-1β or IL-10 were measured during CLP-induced sepsis (^a^P = 0.852 [not significant], ^b^P = 0.002, ^c^P = 0.092 [not significant],^d^P = 0.009, log-rank test; WT [n = 11], WT mice injected with recombinant IL-1β [n = 6] or IL-10 [n = 6], Nod2^−/−^ [n = 10], and Nod2^−/−^ mice injected with recombinant IL-1β [n = 8] or IL-10 [n = 8]). *P<0.05, **P<0.01, ***P<0.001 (one-way ANOVA [b]). Results shown are representative of three independent experiments except for (C) (mean and SEM).

To estimate cytokine-mediated effector functions of immune cells in sepsis, we measured the expression of IL-1β and IL-10 receptors on peritoneal cells. Both IL-1β and IL-10 receptors were similarly expressed on total peritoneal cells of WT and Nod2^−/−^ mice with CLP (Fig. 3A). Next, to determine whether NOD2-mediated IL-1β and IL-10 production plays a critical role in C5a generation during sepsis, we administered rIL-1β or rIL-10 to WT or Nod2^−/−^ mice 4 h or 12 h after CLP, respectively. Administration of rIL-1β or rIL-10 enhanced serum and peritoneal C5a, but not C3a, levels (Fig. 3B). Furthermore, rIL-1β or rIL-10 injection into Nod2^−/−^ mice reduced survival rates during sepsis, whereas these recombinant cytokines did not affect the survival of WT mice (Fig. 3C). These findings indicate that NOD2-mediated IL-1β and IL-10 production by neutrophils contributes to the pathogenesis of sepsis by enhancing C5a generation.

**Figure 4:**
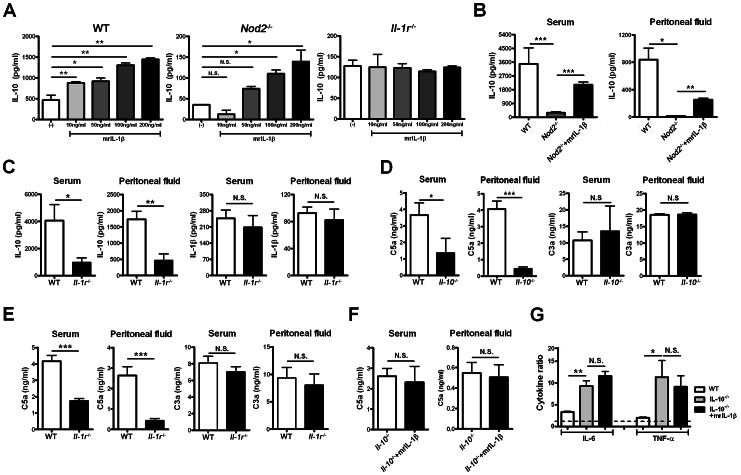
Nucleotide-binding oligomerization domain (NOD)2-mediated IL-1β-dependent IL-10 production by neutrophils enhances C5a generation during sepsis. (A) Peritoneal cells obtained from WT, Nod2^−/−^, or Il-1r^−/−^ mice 12 h after CLP were incubated with recombinant IL-1β for 12 h and IL-10 levels were measured in culture fractions. (B) Serum and peritoneal IL-10 levels were measured in WT, Nod2^−/−^ and Nod2^−/−^ mice injected with recombinant IL-1β 4 h after CLP. (C) Serum and peritoneal IL-10 and IL-1β levels were measured in WT and Il-1r^−/−^ mice 24 h after CLP. (D–F) Serum and peritoneal C5a and C3a levels were measured in WT, Il-10^−/−^ (D), Il-1r^−/−^(E), and Il-10^−/−^ mice injected with recombinant IL-1β 4 h after CLP (F). (G) Recombinant IL-1β was injected into Il-10^−/−^ mice 4 h after CLP. Peritoneal cells obtained from these mice were incubated with or without LPS and cytokine levels were measured. The ratios of individual cytokines (stimulated with LPS/non-stimulated) were estimated. *P<0.05, **P<0.01, ***P<0.001 (one-way ANOVA [a, b, g]), two-tailed unpaired t-test [c–f]) (n = 4 in B–G) Results shown are representative of three independent experiments (mean and SEM).

The IL-1β autocrine loop amplifies the NOD2-mediated induction of pro- and anti-inflammatory cytokines in human monocyte-derived macrophages [23]. Moreover, our experiments and other studies demonstrated that neutrophils were a major subset to produce IL-1β and IL-10 and highly expressed NOD2 during CLP-induced sepsis [24], [25]. These findings led us to hypothesize that IL-1β-dependent IL-10 production by neutrophils may occur in the NOD2-mediated immune response during sepsis. To address this, total peritoneal cells obtained from WT, Nod2^−/−^, or Il-1r^−/−^ mice with CLP were incubated with rIL-1β. IL-10 levels in culture fractions of WT and Nod2-deficient cells were increased in an IL-1β dose-dependent manner, whereas those of Il-1r-deficient cells were not altered (Fig. 4A). Administration of rIL-1β to Nod2^−/−^ mice enhanced serum and peritoneal IL-10 levels during sepsis (Fig. 4B). Furthermore, Il-1r^−/−^ mice showed reduced serum and peritoneal IL-10 levels compared to WT mice with CLP, whereas IL-1β levels were similar (Fig. 4C). These results suggest that peritoneal neutrophils produce IL-10 via IL-1β-dependent signaling during sepsis. Next, to explore whether IL-10 production regulates C3a and C5a levels during sepsis, we measured those levels and survival rates in Il-10^−/−^ and Il-1r^−/−^ mice with CLP. Both groups showed higher survival rates and lower C5a, but not C3a levels than WT mice during sepsis (Fig. S5, Fig. 4D, E). The serum and peritoneal C5a levels were not altered by administration of rIL-1β to Il-10^−/−^ mice (Fig. 4F), whereas injection of recombinant C5a reduced survival rates in Il-10^−/−^ and Il-1r^−/−^ mice during CLP-induced sepsis (Fig. S5). Moreover, rIL-1β did not alter the responsiveness to LPS by peritoneal immune cells from Il-10^−/−^ mice with CLP (Fig. 4G). These findings suggest that NOD2-mediated IL-1β-dependent IL-10 production by neutrophils regulates C5a generation during sepsis, although it is completely ruled out that other peritoneal immune and non-immune cells might contribute to C5a generation.

### NOD2-mediated IL-1β-independent and/or IL-1β-dependent IL-10 production enhances C5a generation by suppressing CD55 expression on neutrophils

**Figure 5:**
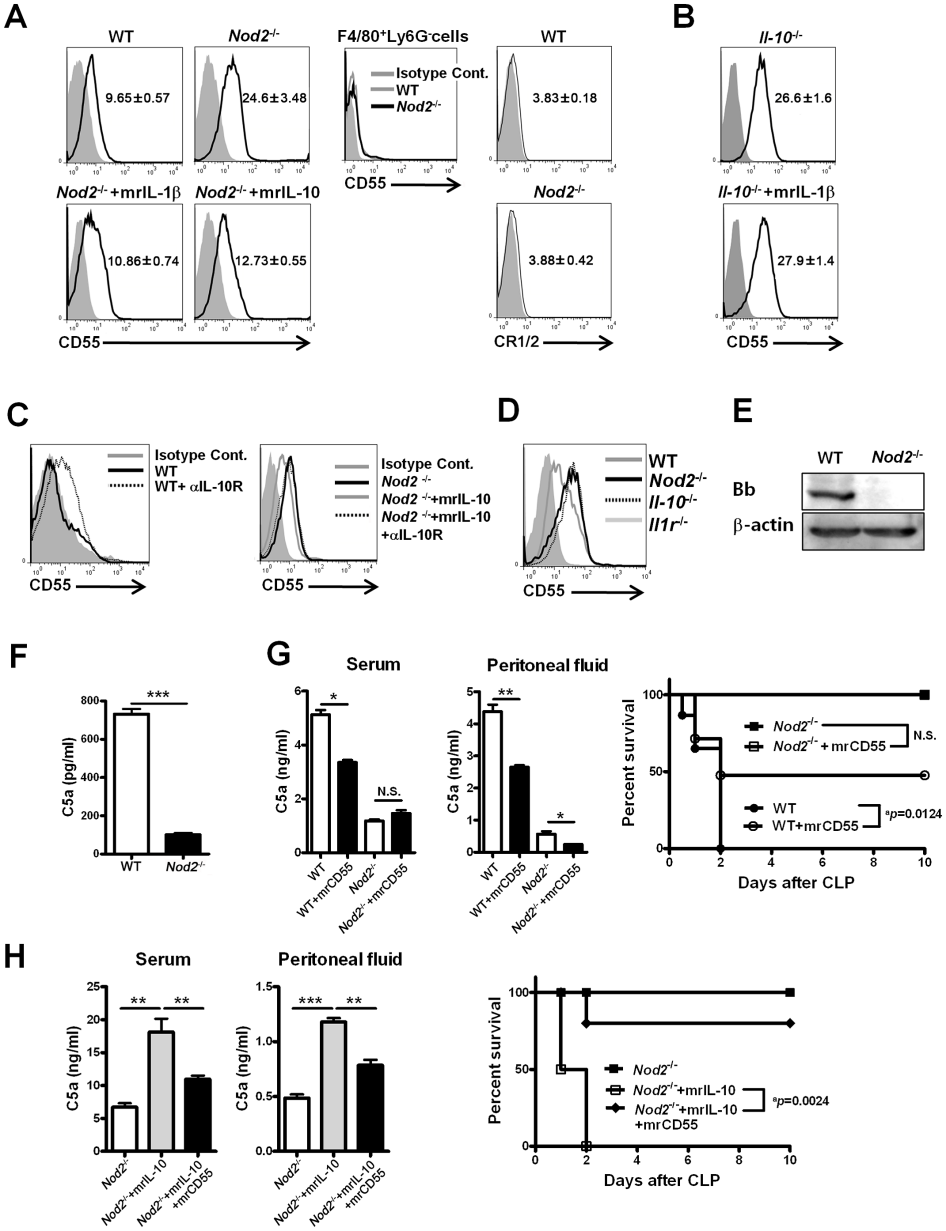
IL-1β-dependent IL-10 production mediated by nucleotide-binding oligomerization domain (NOD) 2 enhances C5a generation by suppressing CD55 expression on Ly6-G^+^ cells during sepsis. (A) CD55 and CR1/2 expression on gated F4/80^−^Ly6-G^+^ peritoneal cells from WT, Nod2^−/−^, and Nod2^−/−^ mice injected with recombinant IL-1β or IL-10 was estimated 24 h after CLP (mean fluorescence intensity [MFI] of CD55 expression in the panels). (B) CD55 expression on gated F4/80^−^Ly6-G^+^ peritoneal cells from Il-10^−/−^ or Il-10^−/−^ mice injected with recombinant IL-1β was estimated 24 h after CLP (MFI of CD55 expression in the panels) (C) To block IL-10 receptor engagement *in vivo*, anti-IL10 receptor mAbs were i.p. injected into WT and Nod2^−/−^ mice administered recombinant IL-10 during CLP-induced sepsis. CD55 expression on gated F4/80^−^Ly6-G^+^ peritoneal cells from these mice 24 h after CLP was evaluated. (D) The levels of CD55 expression on F4/80^−^Ly6-G^+^ peritoneal cells were compared in WT, Nod2^−/−^, IL-10^−/−^, and Il-1r^−/−^ mice 24 h after CLP. (A–D) anti-CD55 mAb (lines) and control IgG (diagrams filled with gray) were used. (E) Peritoneal cells from WT and Nod2^−/−^ mice 24 h after CLP were blotted for Bb factor. (F) Peritoneal cells from WT and Nod2^−/−^ mice 12 h after CLP were incubated with RPMI media containing 10% WT mouse serum for 24 h. (G and H) To evaluate the effect of CD55 on C5a generation *in vivo*, WT, Nod2^−/−^, (G) or Nod2^−/−^ mice given recombinant IL-10 (H) were i.p. injected with soluble CD55 12 h after CLP. Serum and peritoneal C5a levels and the survival percentages of these mice were measured during CLP-induced sepsis (^a^P = 0.0124, log-rank test; WT [n = 8], Nod2^−/−^ [n = 8 ], and soluble CD55-injected WT [n = 6 ] or Nod2^−/−^ mice [n = 8 ] in G, ^a^P = 0.0024, log-rank test; Nod2^−/−^ mice [n = 8], Nod2^−/−^ mice injected with recombinant IL-10 [n = 8] or recombinant IL-10 and soluble CD55 [n = 6] in H). *P<0.05, **P<0.01, ***P<0.001 (two-tailed unpaired t-test [f], one-way ANOVA [g, h]). (n = 3 in A–D, n = 4 in G and H) Results shown are representative of three independent experiments except for survival experiments (mean and SEM).

Several immune molecules, such as CD55 and CR1/2, on the surface of immune cells regulate the complement network by inhibiting complement generation [8]. Thus, to functionally link the expression of these molecules to NOD2-mediated C5a generation during sepsis, CD55 and CR1/2 expression levels on gated peritoneal F4/80^−^Ly-6G^+^ neutrophils and F4/80^+^Ly-6G^−^ macrophages were measured. Peritoneal F4/80^+^Ly-6G^−^ macrophages minimally expressed CD55 and CR1/2 in WT and Nod2^−/−^ mice with CLP (Fig. 5A), suggesting that expression modulation of these molecules on macrophages minimally contributes to NOD2-mediated C5a generation during sepsis. In contrast, CD55 expression levels on peritoneal F4/80^−^Ly-6G^+^ neutrophils from Nod2^−/−^ and Il-10^−/−^ mice were higher than those of WT mice 24 h after CLP, whereas CR1/2 was not detected on peritoneal F4/80^−^Ly-6G^+^ neutrophils from WT and Nod2^−/−^ mice (Fig. 5A, B). Moreover, rIL-10 or rIL-1β administration to Nod2^−/−^ mice decreased CD55 expression on peritoneal F4/80^−^Ly-6G^+^ neutrophils during sepsis (Fig. 5A). However, IL-1β administration to Il-10^−/−^ mice did not decrease CD55 expression on peritoneal F4/80^−^Ly-6G^+^ neutrophils (Fig. 5B). Furthermore, anti-IL-10 receptor mAb increased CD55 expression on peritoneal F4/80^−^Ly-6G^+^ neutrophils of WT and Nod2^−/−^ mice administered rIL-10 (Fig. 5C). These findings suggest that NOD2-mediated IL-1β-dependent IL-10 production decreases CD55 expression on peritoneal neutrophils, which regulates C5a generation during sepsis. However, CD55 expression levels on peritoneal F4/80^−^Ly-6G^+^ neutrophils in Il-1r^−/−^ mice were intermediate between those on cells of WT and Nod2^−/−^ mice with CLP, indicating that NOD2-mediated IL-10 suppresses CD55 expression on peritoneal F4/80^−^Ly-6G^+^ neutrophils in both an IL-1β-dependent, and an IL-1β-independent manner during sepsis (Fig. 5D). In complement system, CD55 inhibits complement convertase activity by dissociating Bb factor from convertase attached on cell membrane [26]. Bb factor expression was minimally detected in Nod2-deficient total peritoneal cells, whereas Bb factor was highly expressed in WT cells (Fig. 5E). Upon incubation with WT mouse serum, total WT peritoneal cells generated more C5a than Nod2-deficient cells (Fig. 5F). These findings suggest that NOD2-mediated suppression of CD55 expression on peritoneal neutrophils enhances C5a generation during sepsis. To confirm this suggestion *in vivo*, soluble CD55 protein was administered to WT, Nod2^−/−^, WT mice depleted neutrophils, or Nod2^−/−^ mice given rIL-10 during sepsis. Soluble CD55 protein decreased serum and peritoneal C5a levels in WT and Nod2^−/−^ mice given rIL-10, resulting in high survival rates (Fig. 5G and H). Neutrophil depletion using anti-Ly-6G mAb increased serum and peritoneal C5a levels in Nod2^−/−^ mice during CLP-induced sepsis, which was reduced by administration of soluble CD 55 (Fig. S6). Taken together, these data suggest that NOD2-mediated IL-1β-dependent and/or IL-1β-independent IL-10 production enhances C5a generation by suppressing CD55 expression on neutrophils, thereby aggravating sepsis.

### Blockade of NOD2 signals attenuates sepsis by suppressing NOD2-mediated IL-1β and IL-10 production, CD55 expression on neutrophils, and C5a generation

**Figure 6:**
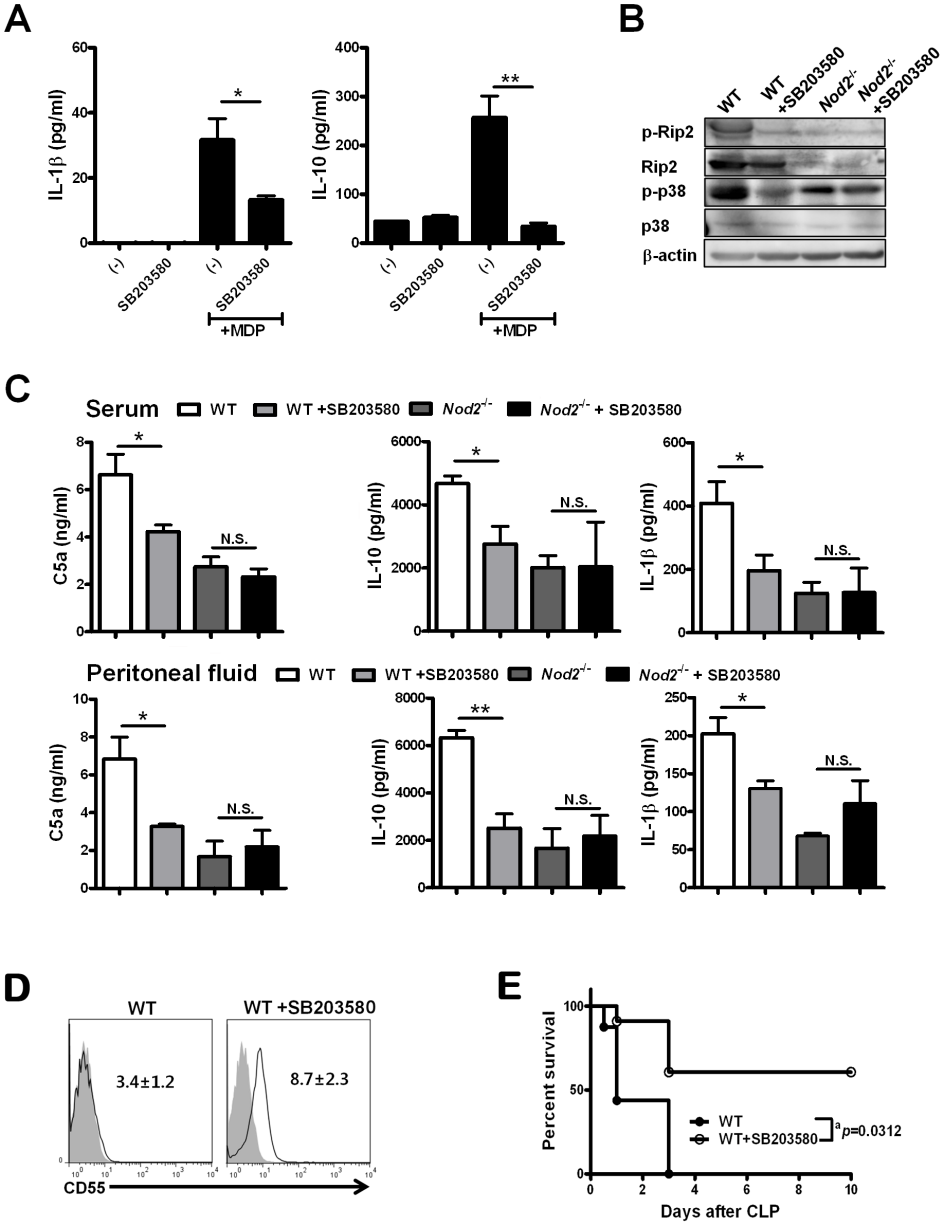
SB203580, an RIP2 inhibitor downstream of nucleotide-binding oligomerization domain (NOD)2, attenuates CLP-induced sepsis. (A) Peritoneal cells of WT mice were cultured with SB203580 and/or MDP for 24 h, and IL-1β and IL-10 concentrations were measured in culture fractions. (B) Molecules related to NOD2-mediated signal transduction were blotted using peritoneal cells obtained from WT and Nod2^−/−^ mice injected with SB203580 or PBS 24 h after CLP. (C) Serum and peritoneal IL-1β, IL-10, and C5a levels were estimated in WT (n = 4) and Nod2^−/−^ (n = 3) mice injected with SB203580 (n = 4 in WT, n = 3 in Nod2^−/−^) or PBS 24 h after CLP by ELISA. (D) The levels of CD55 expression on F4/80^−^Ly6-G^+^ cells from WT (n = 3) and WT mice injected with SB203580 (n = 3) were measured 24 h after CLP. (mean fluorescence intensity [MFI] of CD55 expression in the panels, diagrams filled with gray for istotype-matched control IgG, solid lines for CD55) (E) The percentages of surviving mice were estimated during CLP-induced sepsis (^a^P = 0.0312, log-rank test, n = 6–8 per group; WT mice injected with SB203580 vs. PBS). *P<0.05, **P<0.01, ***P<0.001 (two-tailed unpaired t-test [a, c]). Results shown are representative of three independent experiments except for (E) (mean and SEM).

Upon activation, NOD2 oligomerizes and recruits RIP2 via CARD-CARD interaction, triggering IκB phosphorylation and NF-κB activation [27], [28]. SB203580, an inhibitor of RIP2 and P38 [29], inhibited MDP-mediated IL-1β and IL-10 production by total peritoneal cells from WT mice (Fig. 6A). Furthermore, Nod2^−/−^ mice showed lower levels of RIP2 expression and phosphorylation, and P38 phosphorylation in total peritoneal cells during sepsis than did WT mice (Fig. 6B). Upon SB203580 injection, WT mice exhibited reduced RIP2 expression and phosphorylation, and P38 phosphorylation in total peritoneal cells and serum and peritoneal IL-1β, IL-10, and C5a levels during sepsis, whereas these were unaffected in Nod2^−/−^ mice (Fig. 6B and C). Moreover, SB203580 administration to WT mice increased CD55 expression levels in F4/80^−^Ly-6G^+^ neutrophils, and increased survival rates during sepsis (Fig. 6D and E). These findings suggest that NOD2 blockade inhibits C5a generation by enhancing CD55 expression on neutrophils, depending on IL-1β and IL-10 production by neutrophils and resulting in increased survival rates.

## Discussion

Our experiments demonstrated that serum and peritoneal levels of C5a, but not C3a, were lower in Nod2^−/−^ mice than WT mice during sepsis, while Nod2^−/−^ mice showed higher survival rates than did WT mice, which was reversed by administration of rC5a. NOD2 agonists induce IL-1β production in mice by activating proIL-1β transcription and triggering the release of bioactive IL-1β [30]. Moreover, the 3020insC NOD2 mutant protein in patients with Crohn's disease actively inhibits IL-10 production by impairing hnRNP-A1 phosphorylation and hnRNP-A1 binding to the IL-10 locus [31]. Therefore, it is feasible that NOD2-mediated signals induce IL-1β and IL-10 production by immune cells during sepsis. Consistent with this suggestion, our experiments demonstrated that C5a generation was regulated via NOD2-mediated IL-1β and IL-10 production by peritoneal neutrophils rather than macrophages. This regulation pattern is intriguing in that prototypical pro- and anti-inflammatory cytokines exert a similar effect on C5a generation *in vivo*. Several lines of evidence in our experiments support this regulatory pattern. First, *in vitro* experiments revealed that WT and Nod2-deficient, but not Il-1r-deficient peritoneal cells produced IL-10 in an IL-1β dose-dependent manner. Second, Il-1r^−/−^ mice exhibited lower serum and peritoneal IL-10 and C5a levels during sepsis, although IL-1β levels were similar in Il-1r^−/−^ and WT mice. Third, rIL-1β did not enhance serum and peritoneal C5a levels in Il-10^−/−^ mice, which showed minimal C5a levels compared to WT mice with sepsis, whereas rIL-1β or rIL-10 administration to Nod2^−/−^ mice enhanced serum and peritoneal C5a. Fourth, rIL-1β administration to Nod2^−/−^ mice increased IL-10 and C5a levels during sepsis. Fifth, neutrophil depletion in WT mice reduced the levels of IL-1β, IL-10, and C5a, although these depletion effects were dependent on time points of sepsis. Therefore, the NOD2-mediated IL-1β-IL-10 regulatory loop in neutrophils helps enhancement of C5a generation, which may partially account for the different kinetics of pro- and anti-inflammatory cytokines in sepsis [1], [3]. Furthermore, NOD2-mediated IL-1β and IL-10 production also suppressed LPS-mediated cytokine production by peritoneal immune cells during CLP-induced sepsis (Fig. S7A). Consistent with our results, IL-1 receptor blockade attenuates CLP-induced sepsis [32], [33]. In contrast, injection of human recombinant IL-1α protects against experimental sepsis in a time-dependent manner [34]. Consistent with this suggestion, time points of recombinant IL-1β injection was critical to exert harmful effects on sepsis in Nod2^−/−^ mice (data not shown). Thus, these findings suggest that IL-1 might play diverse functions in sepsis, depending on different time points. Meanwhile, several studies demonstrate that macrophages also produce IL-1β, TNF-α, and IL-6 during sepsis [35], [36]. However, our experiments demonstrated that the levels of IL-6 and TNF-α in serum and peritoneum, and cytosolic IL-1β and IL-10 expression in macrophages were similar between WT and Nod2^−/−^ mice during sepsis. Thus, it is less likely that macrophages might be a main subset to produce IL-1β and IL-10 for regulation of NOD2-mediated C5a generation, although macrophages play a critical role in the regulation of septic responses.

Our experiments demonstrated that NOD2-mediated IL-10 production suppresses CD55 expression on neutrophils in an IL-1β-dependent manner. However, IL-1β regulates CD55 expression only minimally. Furthermore, considering the CD55 expression levels on neutrophils from WT, Il-1r^−/−^, Il-10^−/−^, and Nod2^−/−^ mice with CLP, it is conceivable that NOD2-mediated IL-10 production suppresses CD55 expression on peritoneal neutrophils during sepsis in both IL-1β-dependent and IL-1β-independent manners. Soluble CD55 administration reduced C5a generation and increased the survival rates of WT and Nod2^−/−^ mice injected with rIL-10 during sepsis. Moreover, soluble CD 55 administration also decreased serum and peritoneal C5a levels in Nod2^−/−^ mice depleted neutrophils during CLP-induced sepsis. This suggests that the reduced CD55 expression by neutrophils caused by NOD2-mediated IL-10 production directly regulates C5a generation. This appears to be reasonable because neutrophils represent a major subset of cells in the peritoneum during sepsis. However, the altered CD55 expression on neutrophils affected C5a, but not C3a generation in the NOD2-mediated pathogenesis of sepsis, although CD55 inhibits both C3a and C5a convertase [37]. Thus, it is questionable how reduced CD55 expression on neutrophils inhibits the generation of C5a rather than C3a, in NOD2-mediated pathogenesis of sepsis. CD55 expression on APCs regulates local generation of C5a following cognate interactions between APCs and T cells [38], suggesting that CD55 expressed on immune cells regulates generation of C5a, rather than C3a. Therefore, we postulated that CD55 expressed on neutrophils might inhibit C5a to a greater extent than C3a convertase in the septic microenvironment. Alternatively, compensatory generation of C3a in the complement cascade may account for the relative lack of a change in C3a level during sepsis, even though altered CD55 expression inhibits C3a and C5a convertase equally *in vivo*. Moreover, unknown mechanisms operating during sepsis might explain this unusual situation. To the best of our knowledge, this study provides the first demonstration that IL-1β-dependent and/or IL-1β-independent IL-10 production enhances C5a generation by suppressing CD55 expression on neutrophils during sepsis.

IL-1β and IL-10 concentrations are significantly higher in patients with septic shock than in those with severe sepsis [39]. It generally accepted that IL-1β-mediated systemic inflammatory responses and cardiac dysfunction, and IL-10-mediated immune suppression account for the high mortality in sepsis [40]–[42]. In our experiments, NOD2-mediated IL-1β production exerted not only indirect modulation of C5a generation via the IL-1β-IL-10 loop, but also direct the regulation of septic response by decreasing immune cell phagocytosis and elevated culturable bacterial CFU levels in a C5a- and IL-10-independent manner (Fig. S7B, C). Several studies have reported that IL-10 suppresses immune responses during sepsis by activation-induced apoptosis of T cells, reducing MHC class II expression on APCs, decreasing IFN-γ production, and deactivating monocytes [41]–[43]. However, no differences between WT and Nod2^−/−^ mice were detected in terms of T cell apoptosis and IFN-γ production, whereas the expression levels of MHC class II, CD80, and CD86 on APCs in Nod2^−/−^ mice were lower than those in WT mice during sepsis (Fig. 2A and Figs. S8, S9). Furthermore, administration of rIL-1β or rIL-10 to Nod2^−/−^ mice did not increase CD4^+^ or CD8^+^ T cell apoptosis in the spleen and thymus (Fig. S4). These results suggest that NOD2-mediated IL-10 production minimally modulates T cell apoptosis, activation, and differentiation during sepsis. Therefore, the high mortality of patients during hypo-inflammatory phase of sepsis might be attributable to the effect of IL-10 on both NOD2-mediated C5a generation and immune suppression, which leads to primary and/or secondary hospital-acquired infection [44].

In contrast to protective role in single bacterial infections, NOD2-mediated signals aggravate polymicrobial sepsis. Considering that polymicrobial infection and the septic microenvironment appear to differ from those in monomicrobial infections, it is conceivable that NOD2 plays diverse roles in innate immune responses against bacteria, depending on the *in vivo* microenvironment. Thus, we propose that NOD2 has both protective and provocative functions in immunity to bacterial infection. With regard to NOD2-targeted therapeutics for sepsis associated with bacterium, inhibition of NOD2 signals might be useful. Consistent with this suggestion, our experiments demonstrate that blockade of NOD2 signals via RIP2 and P38 inhibition using SB203580 attenuated sepsis by reducing C5a generation, suggesting that inhibitors of RIP2 and/or its downstream molecules may be therapeutically useful for treatment of patients with sepsis. Moreover, a recent study demonstrated that EGFR tyrosine kinase inhibitors such as gefitinib and erlotinib, already used clinically as chemotherapy for non-small cell lung cancer, inhibited RIP2 tyrosine phosphorylation and MDP-induced cytokine release, but not in an EGFR-dependent manner [45]. Therefore, inhibitors of RIP2 phosphorylation may be effective therapeutic agents against sepsis and NOD2-related immune diseases. However, it has been reported that clinical trials targeting C5a to treat sepsis have failed [46]. Thus, it is need to be circumspect to develop therapeutic approach for sepsis based on NOD2-mediated C5a regulation pathway.

In conclusion, NOD2-mediated signals increase C5a levels by suppressing CD55 expression on neutrophils via IL-1β-dependent or IL-1β-independent IL-10 production by neutrophils, thereby aggravating sepsis.

## Materials and Methods

### Ethics statement

This study was performed in strict accordance with Korean law (ANIMAL PROTECTION LAW). The experimental protocol was approved by the Institutional Animal Care and Use Committee of Biomedical Research Institute of Seoul National University Hospital (SNUH-IACUC No. 12-0130).

### Mice and the cecal ligation and puncture (CLP)-induced sepsis model

Seven- to eight-week-old C57BL/6 mice were purchased from the Orient Company Ltd (Seoul, Korea). Nod2^−/−^, Il-10^−/−^, and Il-1r^−/−^ mice were purchased from the Jackson Laboratory (Bar Harbor, ME, USA). The mice were bred and maintained under specific pathogen-free conditions at the Biomedical Research Institute Seoul National University Hospital. To perform CLP-induced sepsis, the mouse cecum was exposed through an 1 cm incision, and the cecum was ligated below the ileocecal valve using a 5-0 Ethilon suture (Ethicon, Somerville, NJ, USA) without causing bowel obstruction. Then the cecum was punctured with a 26-gauge needle at two different spots. In neutrophils depletion experiments using anti-Ly-6G antibody *in vivo*, the cecum was punctured at one spot.

### Injection of mice with reagents

Mouse recombinant (mr) IL-1β 40 µg/mouse) or IL-10 (30 µg/mouse) (ProSpec-Tany TechnoGene, Rehovot, Israel) in PBS was i.p. injected into mice 4 or 12 h after CLP, respectively. mrC5a (5 µg/injection) (R&D Systems Inc., Minneapolis, MN, USA) was i.p. injected into mice 4 and 12 h after CLP. To block IL-10 signaling *in vivo*, WT mice were intravenously injected anti-IL-10R mAb (200 µg/mouse) (BD Bioscience, Sparks, MD), 1 day prior to CLP. mrCD55 20 µg/mouse) (R&D systems) was i.p. injected into mice 12 h after CLP. To deplete neutrophils *in vivo*, WT and Nod2^−/−^ mice were i.p. injected with anti-Ly-6G antibody (150 µg/mouse) (Bioledgend, San Diego, CA, USA) 0 and 6 h after CLP. SB203508 (0.1 µmol/mouse) (Sigma Aldrich) in 200 µl 0.5% PBST was i.p. injected into WT mice 1, 5, 16 h after CLP.

### Bacterial CFU counts

Bacterial CFUs were counted by plating serial dilutions of blood and liver homogenates onto blood agar plates (Hanil Komed, Seoul, Korea), which were incubated in 5% CO_2_ at 37°C overnight. The number of colonies was counted after incubation for 18 h.

### Cell preparation and culture

To obtain total peritoneal cells, we injected 4 ml of RPMI media containing 2.5% FBS into peritoneal cavity of mice and collected cells from peritoneal fluids. Peritoneal cells (5×10^5^) from mice were cultured with MDP (20 µg/ml) (Sigma Aldrich) in the presence or absence of SB203580 (100 nM) (Calbiochem, San Diego, CA.) for 20–24 h. To estimate the responsiveness of immune cells to LPS, cells (5×10^5^) were cultured with LPS (1 µg/ml) for 7 h and various cytokine concentrations were measured.

### ELISA

All cytokines and complements were measured using a BD Bioscience ELISA kit according to the manufacturer's instructions. ELISA assay for C5a is not detecting C5.

### Flow cytometric analysis and intracellular staining

Cells were incubated with antibodies on ice for 30 min in 100 µl staining buffer (0.5% BSA). FITC- or PE Cy7-conjugated anti-Ly-6G, PE-conjugated anti-CD55, anti-IL-10R, anti-IL-1R, anti-CD4, anti-CD8, anti-MHC class II mAb, FITC-conjugated anti-CD80, anti-CD86, anti-annexin V mAbs, and 7-amino-actinomycin D were purchased from BD Biosciences. A PE Cy5- or Alexa 647 (eBioscience, San Diego, CA, USA)-conjugated anti-F4/80 mAb was used. CD55 expression was estimated on gated F4/80^+^Ly-6G^−^ and F4/80^−^Ly-6G^+^ peritoneal cells. To perform intracellular staining, peritoneal cells were obtained from mice 4 h and 12 h after CLP and incubated 4 h with 1 µl/ml GolgiStop (BD Bioscience). Cells were surface stained with Cy7-conjugated anti-Ly-6G and Alexa 647-conjugated anti-F4/80 mAb, and incubated with fixation/permeabilization solution of Cytofix/Cytoperm Kit (BD Biosciences) for 20 min. After washing, the cells were stained with PE-conjugated anti-IL-10 or FITC-conjugated anti-IL-1βmAb (BD Biosciences). Cells were run on an FACS LSR II or FACS caliber (BD Bioscience), and analyzed using the Flowjo software (Treestar, Ashland, OR, USA).

### Peritoneal fluid cell sorting

Peritoneal fluid cells obtained from mice were stained with FITC-conjugated anti-Ly-6G mAb (BD Biosciences) and PE Cy5-conjugated anti-F4/80 mAb (eBioscience). Then, stained cells were sorted on a BD FACSAria flow cytometer (Franklin Lakes, NJ, USA). Sorted Ly-6G^+^F4/80^−^ and Ly-6G^−^F480^+^ cells were isolated at 98% purity.

### Real-time PCR analysis

An RNeasy Mini kit (Qiagen, Courtaboeuf, France) was used to isolate mRNA from sorted peritoneal Ly-6G^+^F4/80^−^ and Ly-6G^−^F480^+^ cells. RNA (3 µg) was reverse-transcribed into cDNA using M-MLV Reverse Transcriptase (Promega, Madison, WI, USA). PCR was performed using cDNA as a template with primers and probes from Applied Biosystems (Foster City, CA, USA) and Biosource (Camarillo, CA, USA) for GAPDH, NOD2, IL-1β, and IL-10 (TaqMan pre-developed Assay Reagent). Gene expression levels were normalized to that of GAPDH.

### Measurement of bacterial phagocytosis by peritoneal immune cells

Mouse peritoneal cells were cultured with FITC-labeled *E. coli* (Invitrogen, Carlsbad, CA, USA) for 15 min. Attached cells were washed with warm PBS three times and then treated with 0.2% trypan blue for 1 min at room temperature. Then the cells were fixed with 4% formalin for 15 min and cultured with FITC-labeled *E. coli* to estimate nonspecific binding of *E. coli* to the cell surface. These cells, which were cultured with FITC-labeled *E. coli*, were run on the FACs caliber and analyzed with the Flowjo software.

### Western blotting

Western blotting was performed as described previously [47]. Antibodies against phosphor–p38, p38 (Cell Signaling Technology, MA, USA), phosphor-Rip2 (Thermo scientific, Rockford, USA), Rip2 (Santa Cruz Biotechnology, CA, USA), Bb (Santa Cruz Biotechnology), NOD2 (Santa Cruz Biotechnology), and a horseradish peroxidase-conjugated goat anti-rabbit IgG (Thermo scientific) were used.

### Statistical analysis

Survival data were plotted as Kaplan-Meier survival curves and analyzed using the log-rank test. Statistical significance was analyzed using Prism ver. 5.0 (GraphPad Software Inc., San Diego, CA, USA). One-way and two-way analyses of variance (ANOVA) and t-tests were performed, and a post hoc test was used if P<0.05. Data are expressed as the mean ± standard error of the mean (SEM).

## Supporting Information

Figure S1 The intestinal bacterial profile of WT and nucleotide-binding oligomerization domain (Nod)2^−/−^ mice constitutes only a minimal contribution to the NOD2-mediated regulation of CLP-induced sepsis. (A) Cecum contents from WT (left panel) or Nod2^−/−^ (right panel) mice were injected into WT or Nod2^−/−^ mice that had undergone cecum ligation without puncture, and survival rates were determined. (B) Cohoused WT and Nod2^−/−^ mice for 4 weeks were estimated for survival rates during CLP-induced sepsis. (^a^P = 0.002, ^b^P = 0.0308, ^c^P = 0.0164, log-rank test, n = 6 per group; WT vs. Nod2^−/−^ mice).
(TIF)

Figure S2 Neutrophils and macrophages similarly infiltrate peritoneum of WT and nucleotide-binding oligomerization domain (Nod)2^−/−^ mice during CLP-induced sepsis. The percentages of F4/80^−^Ly-6G^+^ and F4/80^+^Ly-6G^−^ peritoneal cells from WT (n = 3) and Nod2^−/−^ (n = 3) mice before, 4, 12, and 24 h after CLP were analyzed using flow cytometry. Results shown are representative of three independent experiments.
(TIF)

Figure S3 The expression pattern of nucleotide-binding oligomerization domain (NOD)2 in peritoneal cells during cecal ligation and puncture (CLP)-induced sepsis. (A) NOD2 expression was estimated in peritoneal cells from WT and Nod2^−/−^ mice 4–6 h after thioglycollate injection by Western blot. (B) The NOD2 expression pattern was estimated in sorted F4/80^−^Ly-6G^+^ and F4/80^+^Ly-6G^−^ peritoneal cells of WT mice 4, 12, and 24 h after CLP by Western blot.
(TIF)

Figure S4 Neutrophils produce IL-1β and IL-10 during cecal ligation and puncture (CLP)-induced sepsis. (A) F4/80^−^Ly-6G^+^ and F4/80^+^Ly-6G^−^ cells were obtained from WT and nucleotide-binding oligomerization domain (Nod)2^−/−^ mice 4 h after CLP, sorted, and cultured 24 h without stimulation. IL-1β and IL-10 levels in culture supernatant were measured using ELISA. (B and C) Anti-Ly-6G mAb was injected into WT mice 0 and 6 h after CLP. (B) Flow cytometric analysis shows Ly-6G^+^ cells in WT mice before and after injection of anti-Ly-6G mAb. (C) The levels of IL-1β and IL-10 in serum and peritoneum of WT mice injected with anti-Ly-6G mAb or PBS by ELISA. *P<0.05, **P<0.01, ***P<0.001 (two-tailed unpaired t-test [a]) (one-way ANOVA [c]). (n = 5 in C) Results shown are representative of three or two independent experiments (mean and SEM).
(TIF)

Figure S5 Il-10^−/−^ and Il-1r^−/−^ mice show higher survival rates than WT mice during cecal ligation and puncture(CLP)-induced sepsis. Il-10^−/−^ and Il-1r^−/−^ mice were i.p. injected with mouse recombinant (mr) C5a. The percentages of surviving mice were estimated during CLP-induced sepsis. (^a^P<0.001, ^b^P = 0.0052, ^c^P<0.001, ^d^P = 0.05, log-rank test, n = 8 per each group; WT vs Il-1r^−/−^ mice or Il-10^−/−^, Il-1r^−/−^ mice vs Il-1r^−/−^ mice injected with mrC5a and Il-10^−/−^ vs Il-10^−/−^ mice injected with mrC5a).
(TIF)

Figure S6 Recombinant CD55 reduces C5a levels in nucleotide-binding oligomerization domain (Nod)2^−/−^ mice depleted neutrophils during cecal ligation and puncture (CLP)-induced sepsis. (A) To deplete neutrophils, anti-Ly-6G mAb was injected into Nod2^−/−^ mice 6 h after CLP. The levels of C5a were measured by ELISA in serum and peritoneum of Nod2^−/−^, Nod2^−/−^ mice depleted neutrophils, and Nod2^−/−^ mice depleted neutrophils and administered mouse recombinant CD55. *P<0.05, **P<0.01, ***P<0.001 (one-way ANOVA). (n = 5 in each group) Results shown are representative of two independent experiments (mean and SEM).
(TIF)

Figure S7 LPS-mediated cytokine production by peritoneal cells is suppressed by nucleotide-binding oligomerization domain (NOD2)-mediated IL-1β and IL-10, while phagocytosis is also decreased by NOD2-mediated IL-1β during sepsis. (A) Peritoneal cells obtained from WT, Nod2^−/−^, and Nod2^−/−^ mice injected with recombinant IL-1β or IL-10 were incubated with LPS or PBS for 6 h, and cytokine levels were measured. The ratios of individual cytokines were determined by estimating cytokine levels in LPS vs. PBS culture supernatant fractions. (B) The phagocytic activity of peritoneal cells from these mice was determined by measuring the percentages of cells with intracellular FITC-conjugated *E. coli* after 15 min incubation. (C) Culturable bacterial CFUs were estimated using blood and liver homogenates obtained from WT and Nod2^−/−^ mice 24 h after CLP. *P<0.05, **P<0.01, ***P<0.001 (one-way ANOVA [a, c]). (n = 4 in A–C) Results shown are representative of three independent experiments (mean and SEM).
(TIF)

Figure S8 T cell apoptosis in the spleen and thymus is similar in WT and nucleotide-binding oligomerization domain (Nod)2^−/−^ mice during cecal ligation and puncture (CLP)-induced sepsis. Cells obtained from the spleen and thymus of WT B6 or Nod2^−/−^ mice 24 h after CLP were stained for flow cytometric analysis. Gated CD8^+^ and CD4^+^ T cells were plotted for 7AAD and annexin V. Numbers in diagrams represent the percentages of cells positive for the molecules indicated. (n = 3) Results shown are representative of three independent experiments. N.S.; not significant (one-way ANOVA).
(TIF)

Figure S9 Expression levels of MHC class II, CD80, and CD86 on F4/80^+^Ly-6G^−^ and F4/80^−^Ly-6G^+^ peritoneal cells are similar in WT and nucleotide-binding oligomerization domain (Nod)2^−/−^ mice following cecal ligation and puncture (CLP). Expression levels were estimated on gated F4/80^+^Ly-6G^−^ and F4/80^−^Ly-6G^+^ peritoneal cells obtained from WT B6 or Nod2^−/−^ mice 24 h after CLP. Numbers in diagrams represent mean fluorescence intensity (MFI) for the molecules indicated. (n = 3) Results shown are representative of three independent experiments. N.S.; not significant (one-way ANOVA).
(TIF)
