# Supplementary figures and images for: NOD2-mediated Suppression of CD55 on Neutrophils Enhances C5a Generation During Polymicrobial Sepsis
Source: PLoS Pathog. 2013 May 9;9(5):e1003351. doi: 10.1371/journal.ppat.1003351 (PMC3649968; doi:10.1371/journal.ppat.1003351)

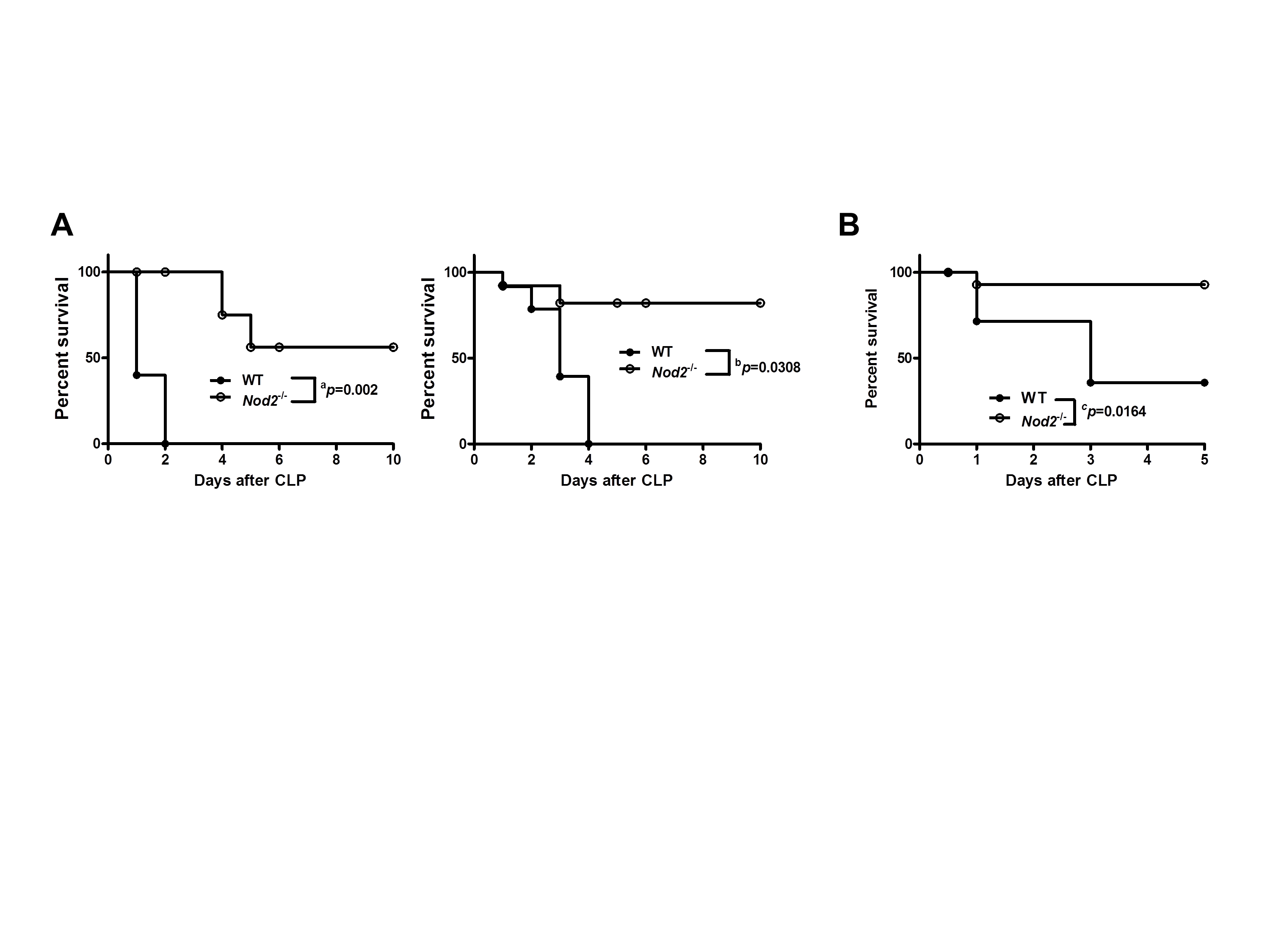

Supplement: Figure S1 — The intestinal bacterial profile of WT and nucleotide-binding oligomerization domain (Nod)2−/− mice constitutes only a minimal contribution to the NOD2-mediated regulation of CLP-induced sepsis. (A) Cecum contents from WT (left panel) or Nod2−/− (right panel) mice were injected into WT or Nod2−/− mice that had undergone cecum ligation without puncture, and survival rates were determined. (B) Cohoused WT and Nod2−/− mice for 4 weeks were estimated for survival rates during CLP-induced sepsis. (aP = 0.002, bP = 0.0308, cP = 0.0164, log-rank test, n = 6 per group; WT vs. Nod2−/− mice). (TIF) [file ppat.1003351.s001.tif]

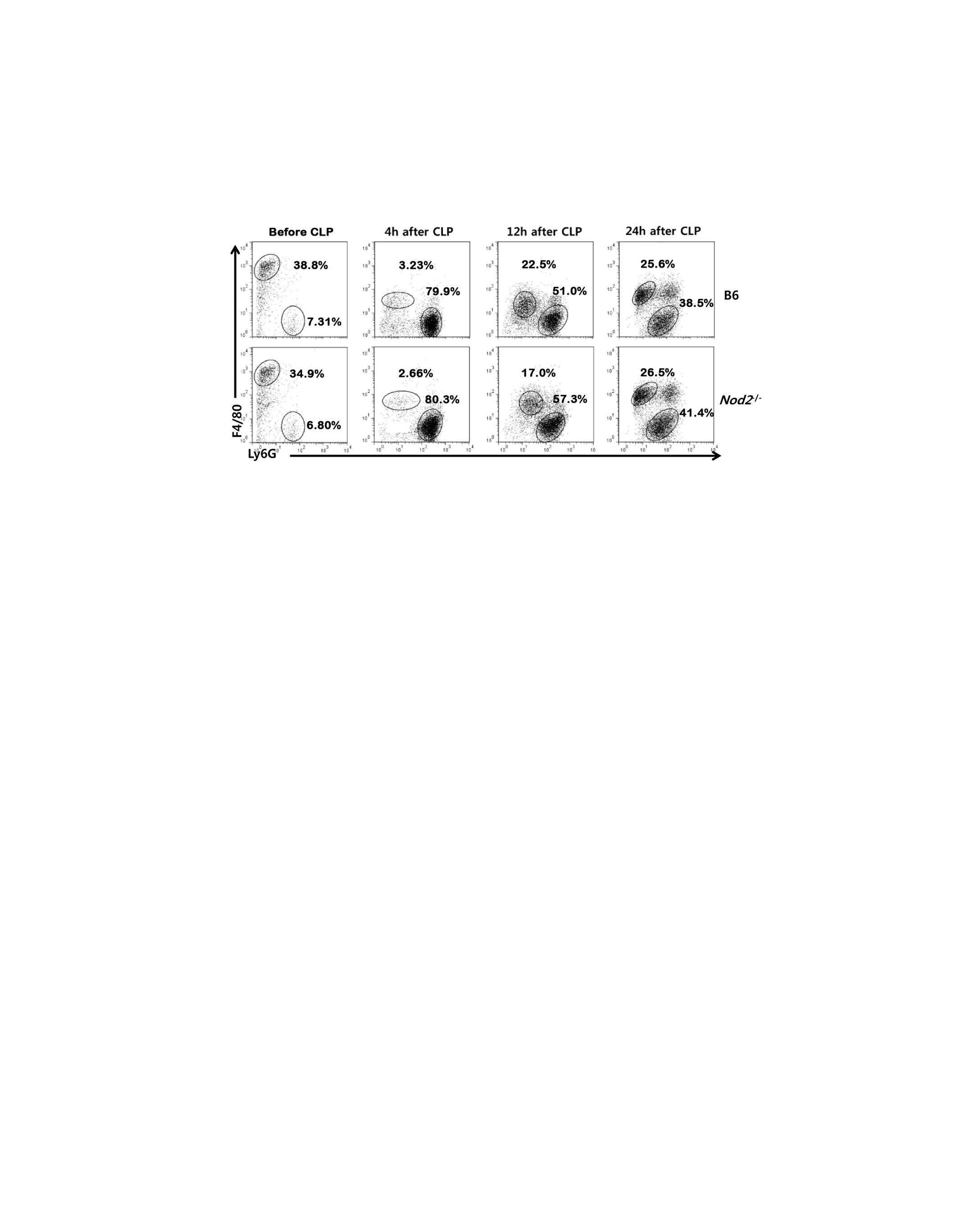

Supplement: Figure S2 — Neutrophils and macrophages similarly infiltrate peritoneum of WT and nucleotide-binding oligomerization domain (Nod)2−/− mice during CLP-induced sepsis. The percentages of F4/80−Ly-6G+ and F4/80+Ly-6G− peritoneal cells from WT (n = 3) and Nod2−/− (n = 3) mice before, 4, 12, and 24 h after CLP were analyzed using flow cytometry. Results shown are representative of three independent experiments. (TIF) [file ppat.1003351.s002.tif]

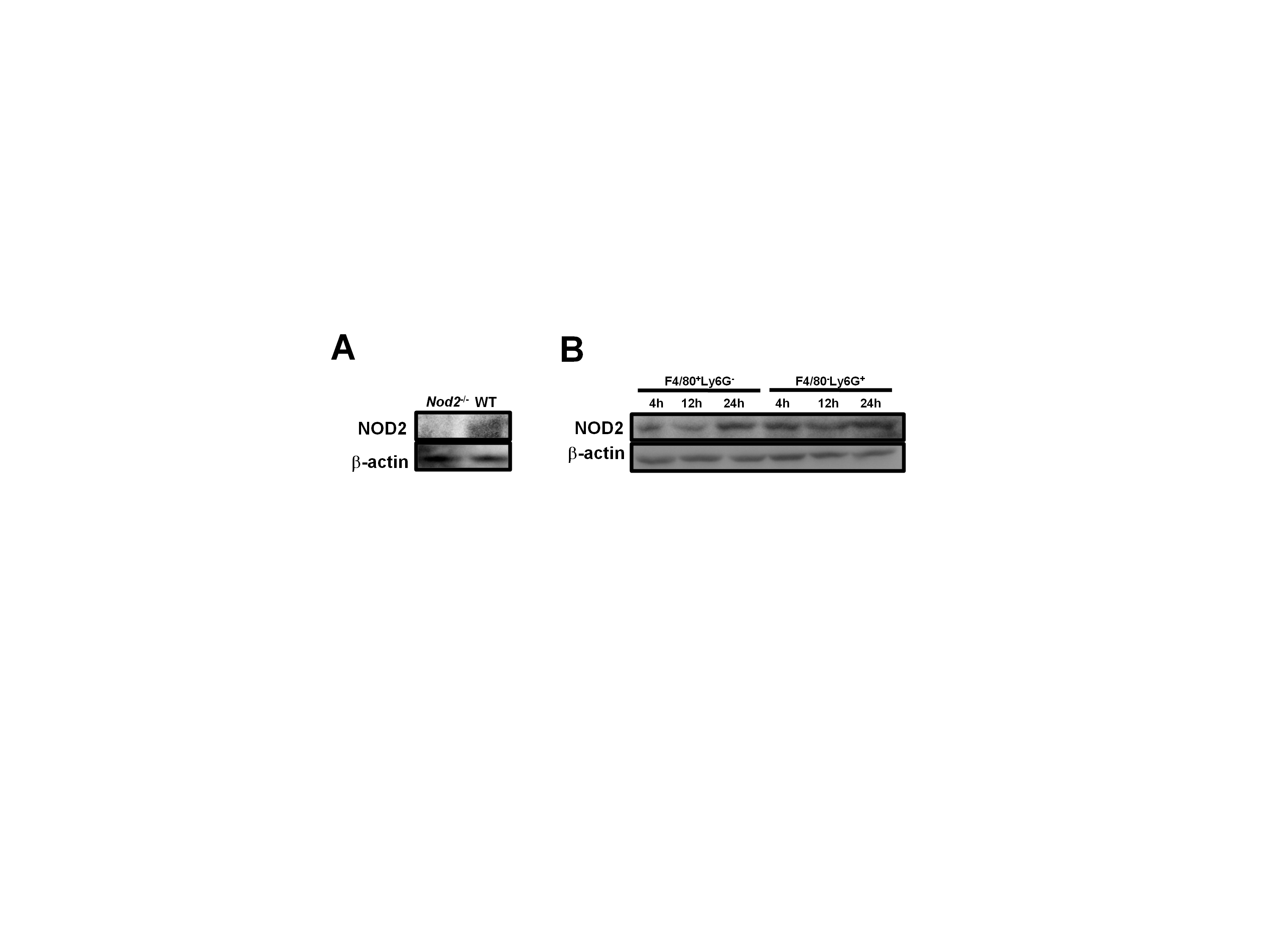

Supplement: Figure S3 — The expression pattern of nucleotide-binding oligomerization domain (NOD)2 in peritoneal cells during cecal ligation and puncture (CLP)-induced sepsis. (A) NOD2 expression was estimated in peritoneal cells from WT and Nod2−/− mice 4–6 h after thioglycollate injection by Western blot. (B) The NOD2 expression pattern was estimated in sorted F4/80−Ly-6G+ and F4/80+Ly-6G− peritoneal cells of WT mice 4, 12, and 24 h after CLP by Western blot. (TIF) [file ppat.1003351.s003.tif]

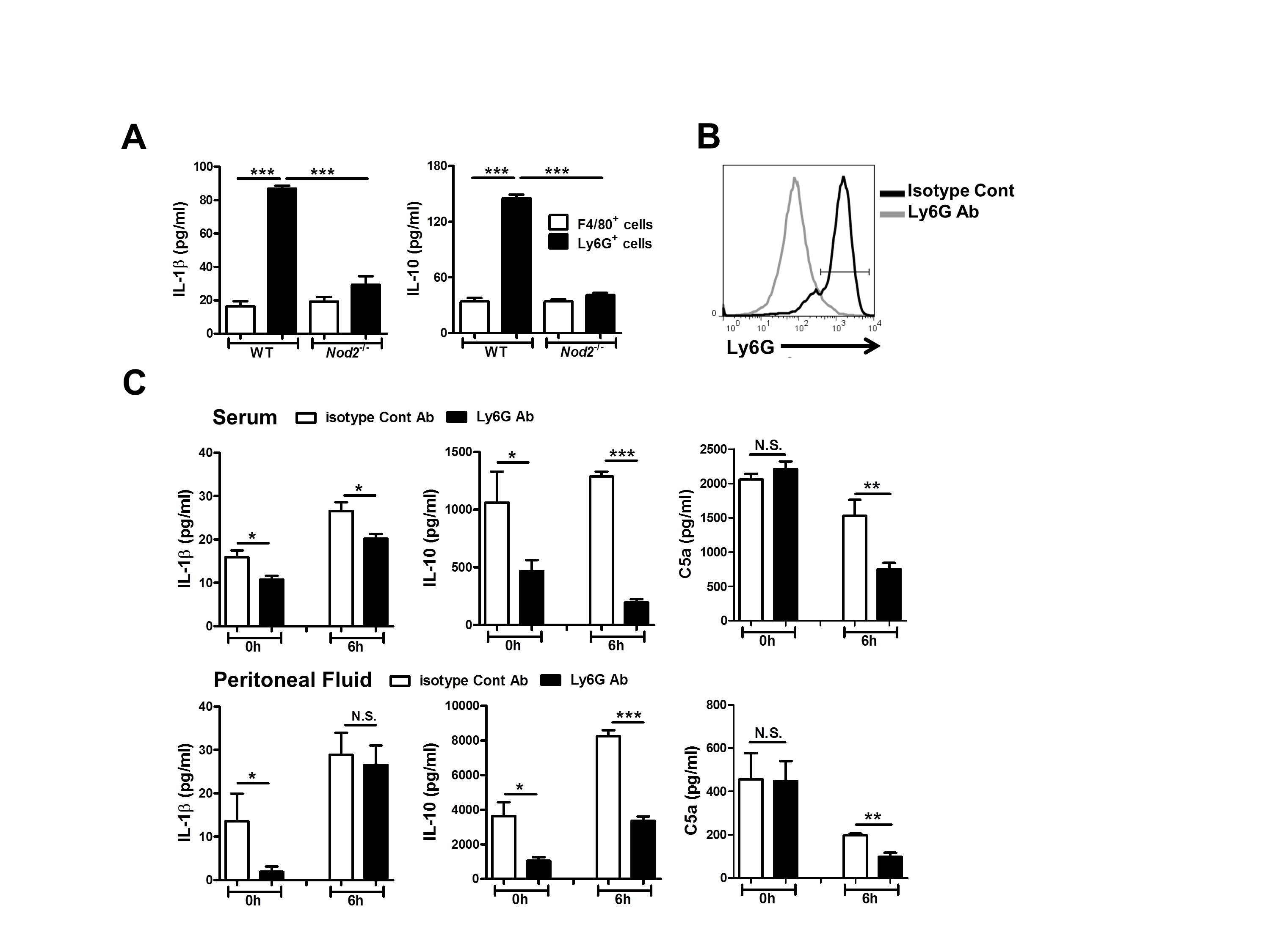

Supplement: Figure S4 — Neutrophils produce IL-1β and IL-10 during cecal ligation and puncture (CLP)-induced sepsis. (A) F4/80−Ly-6G+ and F4/80+Ly-6G− cells were obtained from WT and nucleotide-binding oligomerization domain (Nod)2−/− mice 4 h after CLP, sorted, and cultured 24 h without stimulation. IL-1β and IL-10 levels in culture supernatant were measured using ELISA. (B and C) Anti-Ly-6G mAb was injected into WT mice 0 and 6 h after CLP. (B) Flow cytometric analysis shows Ly-6G+ cells in WT mice before and after injection of anti-Ly-6G mAb. (C) The levels of IL-1β and IL-10 in serum and peritoneum of WT mice injected with anti-Ly-6G mAb or PBS by ELISA. *P<0.05, **P<0.01, ***P<0.001 (two-tailed unpaired t-test [a]) (one-way ANOVA [c]). (n = 5 in C) Results shown are representative of three or two independent experiments (mean and SEM). (TIF) [file ppat.1003351.s004.tif]

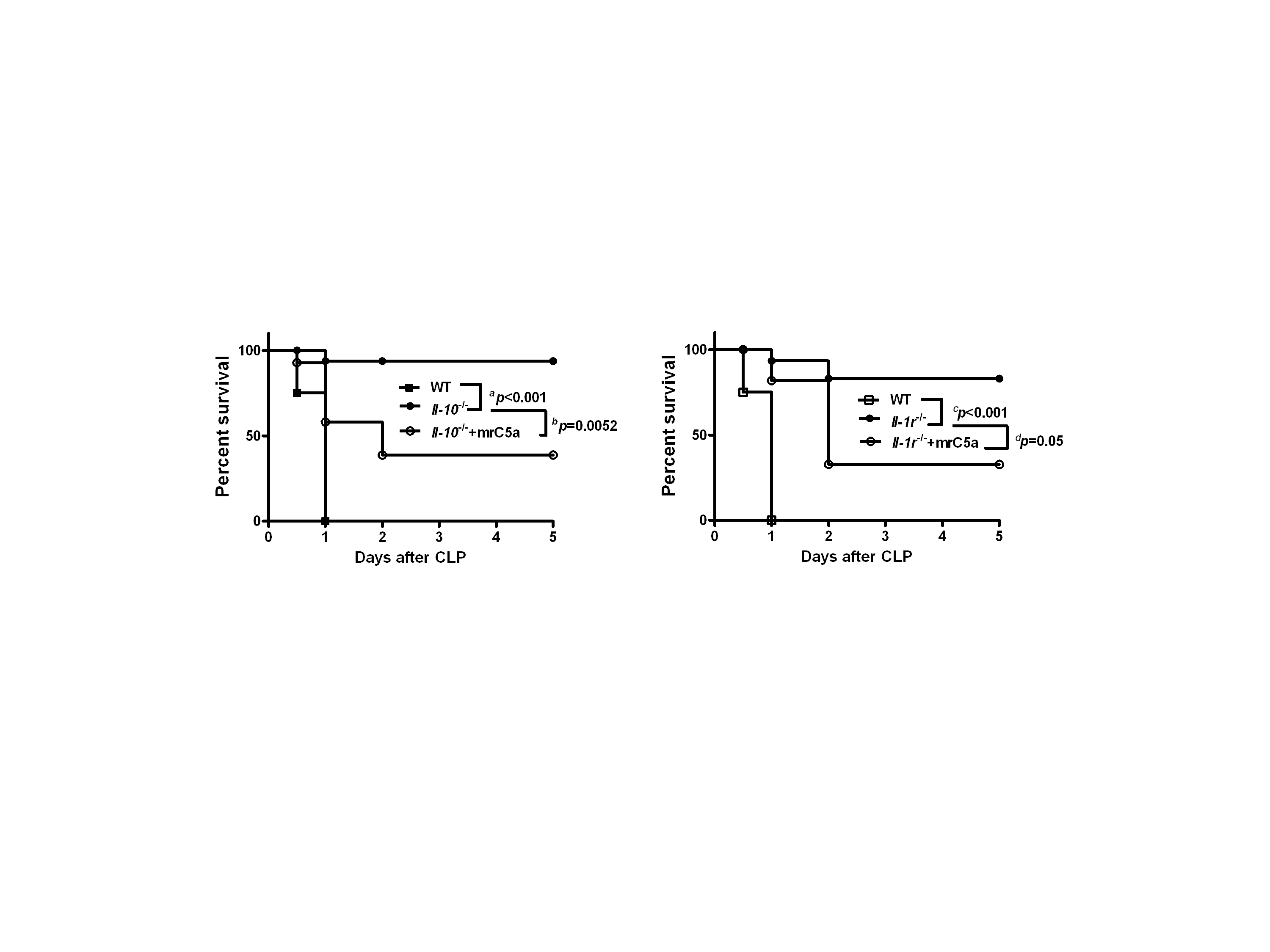

Supplement: Figure S5 — Il-10−/− and Il-1r−/− mice show higher survival rates than WT mice during cecal ligation and puncture(CLP)-induced sepsis. Il-10−/− and Il-1r−/− mice were i.p. injected with mouse recombinant (mr) C5a. The percentages of surviving mice were estimated during CLP-induced sepsis. (aP<0.001, bP = 0.0052, cP<0.001, dP = 0.05, log-rank test, n = 8 per each group; WT vs Il-1r−/− mice or Il-10−/−, Il-1r−/− mice vs Il-1r−/− mice injected with mrC5a and Il-10−/− vs Il-10−/− mice injected with mrC5a). (TIF) [file ppat.1003351.s005.tif]

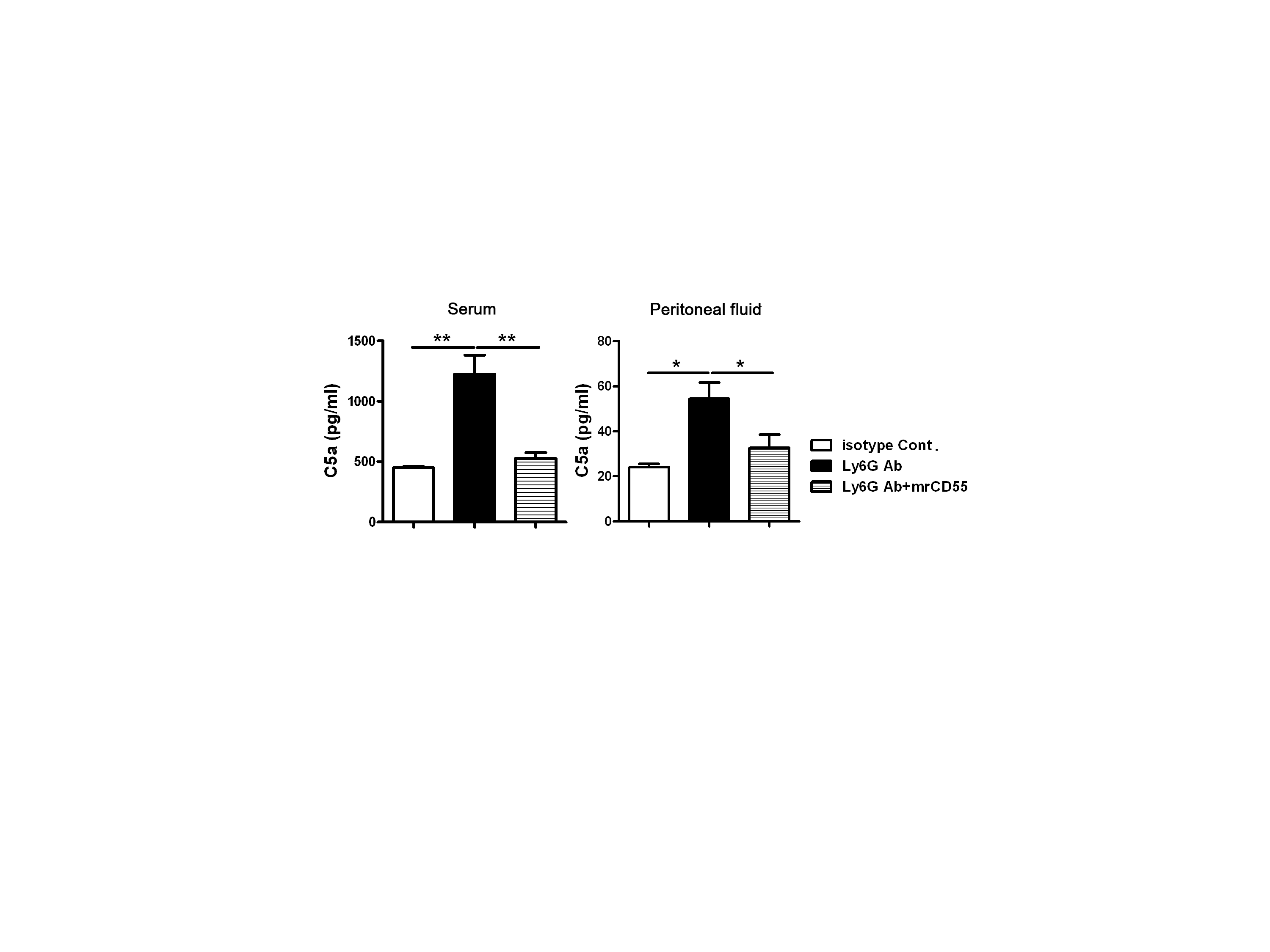

Supplement: Figure S6 — Recombinant CD55 reduces C5a levels in nucleotide-binding oligomerization domain (Nod)2−/− mice depleted neutrophils during cecal ligation and puncture (CLP)-induced sepsis. (A) To deplete neutrophils, anti-Ly-6G mAb was injected into Nod2−/− mice 6 h after CLP. The levels of C5a were measured by ELISA in serum and peritoneum of Nod2−/−, Nod2−/− mice depleted neutrophils, and Nod2−/− mice depleted neutrophils and administered mouse recombinant CD55. *P<0.05, **P<0.01, ***P<0.001 (one-way ANOVA). (n = 5 in each group) Results shown are representative of two independent experiments (mean and SEM). (TIF) [file ppat.1003351.s006.tif]

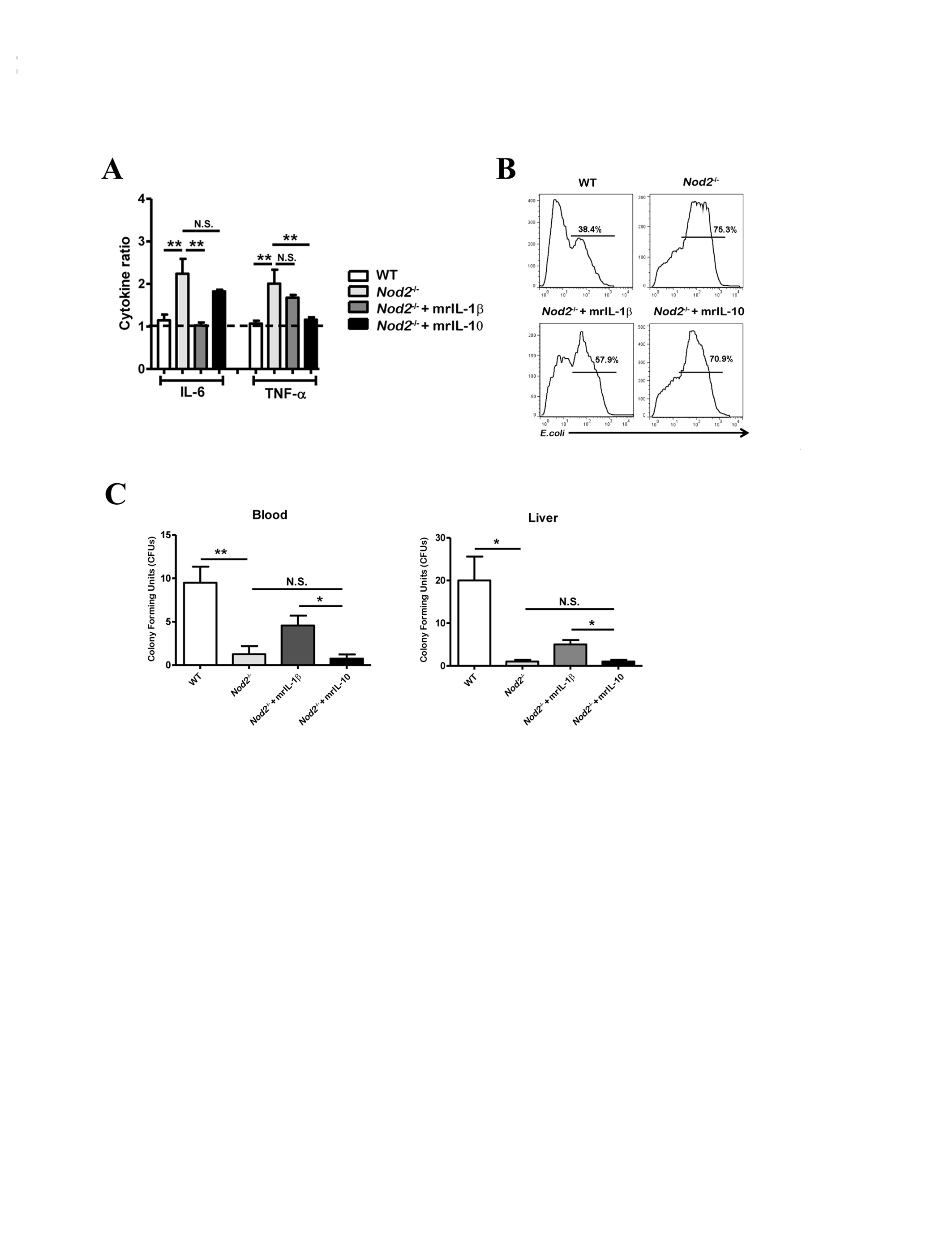

Supplement: Figure S7 — LPS-mediated cytokine production by peritoneal cells is suppressed by nucleotide-binding oligomerization domain (NOD2)-mediated IL-1β and IL-10, while phagocytosis is also decreased by NOD2-mediated IL-1β during sepsis. (A) Peritoneal cells obtained from WT, Nod2−/−, and Nod2−/− mice injected with recombinant IL-1β or IL-10 were incubated with LPS or PBS for 6 h, and cytokine levels were measured. The ratios of individual cytokines were determined by estimating cytokine levels in LPS vs. PBS culture supernatant fractions. (B) The phagocytic activity of peritoneal cells from these mice was determined by measuring the percentages of cells with intracellular FITC-conjugated E. coli after 15 min incubation. (C) Culturable bacterial CFUs were estimated using blood and liver homogenates obtained from WT and Nod2−/− mice 24 h after CLP. *P<0.05, **P<0.01, ***P<0.001 (one-way ANOVA [a, c]). (n = 4 in A–C) Results shown are representative of three independent experiments (mean and SEM). (TIF) [file ppat.1003351.s007.tif]

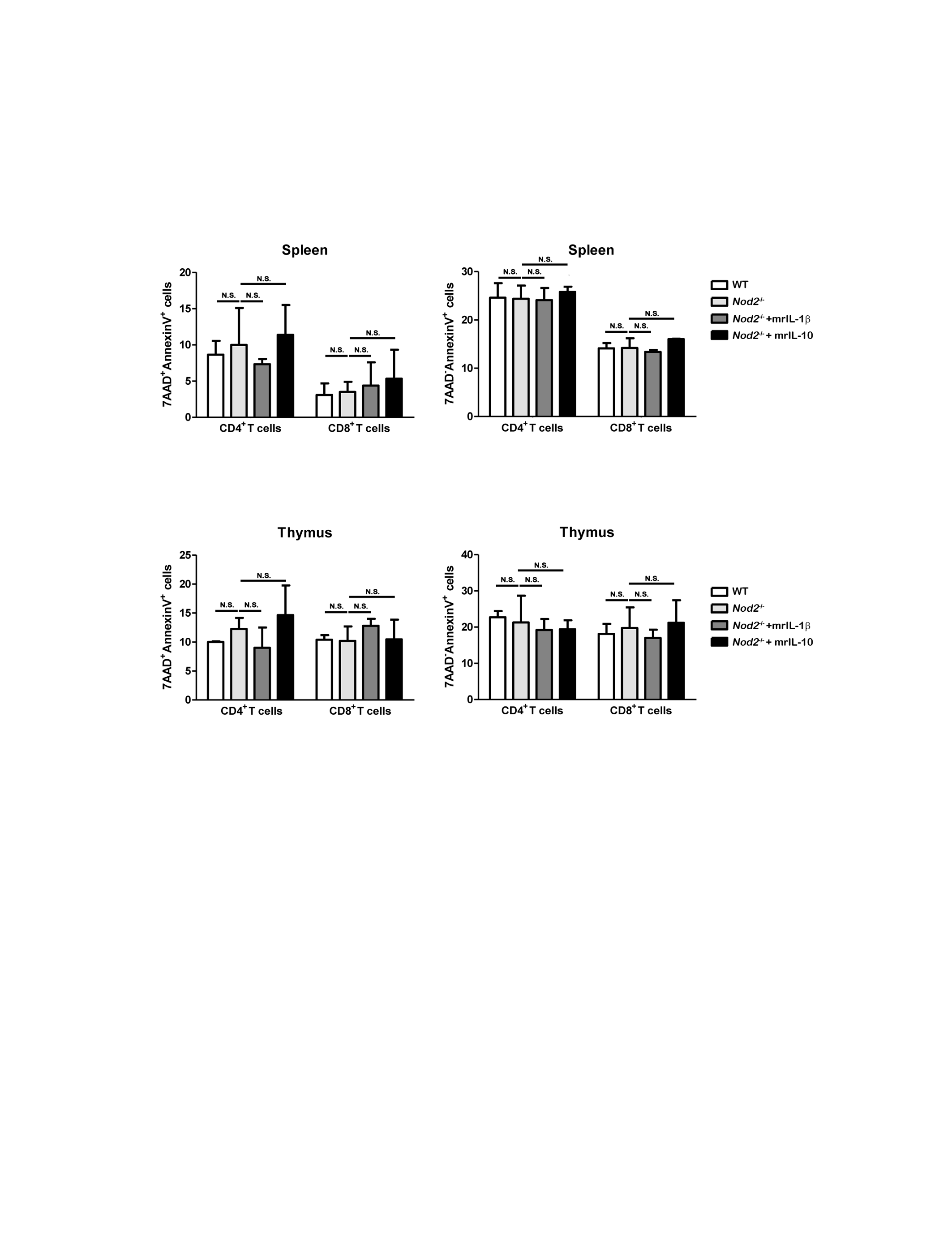

Supplement: Figure S8 — T cell apoptosis in the spleen and thymus is similar in WT and nucleotide-binding oligomerization domain (Nod)2−/− mice during cecal ligation and puncture (CLP)-induced sepsis. Cells obtained from the spleen and thymus of WT B6 or Nod2−/− mice 24 h after CLP were stained for flow cytometric analysis. Gated CD8+ and CD4+ T cells were plotted for 7AAD and annexin V. Numbers in diagrams represent the percentages of cells positive for the molecules indicated. (n = 3) Results shown are representative of three independent experiments. N.S.; not significant (one-way ANOVA). (TIF) [file ppat.1003351.s008.tif]

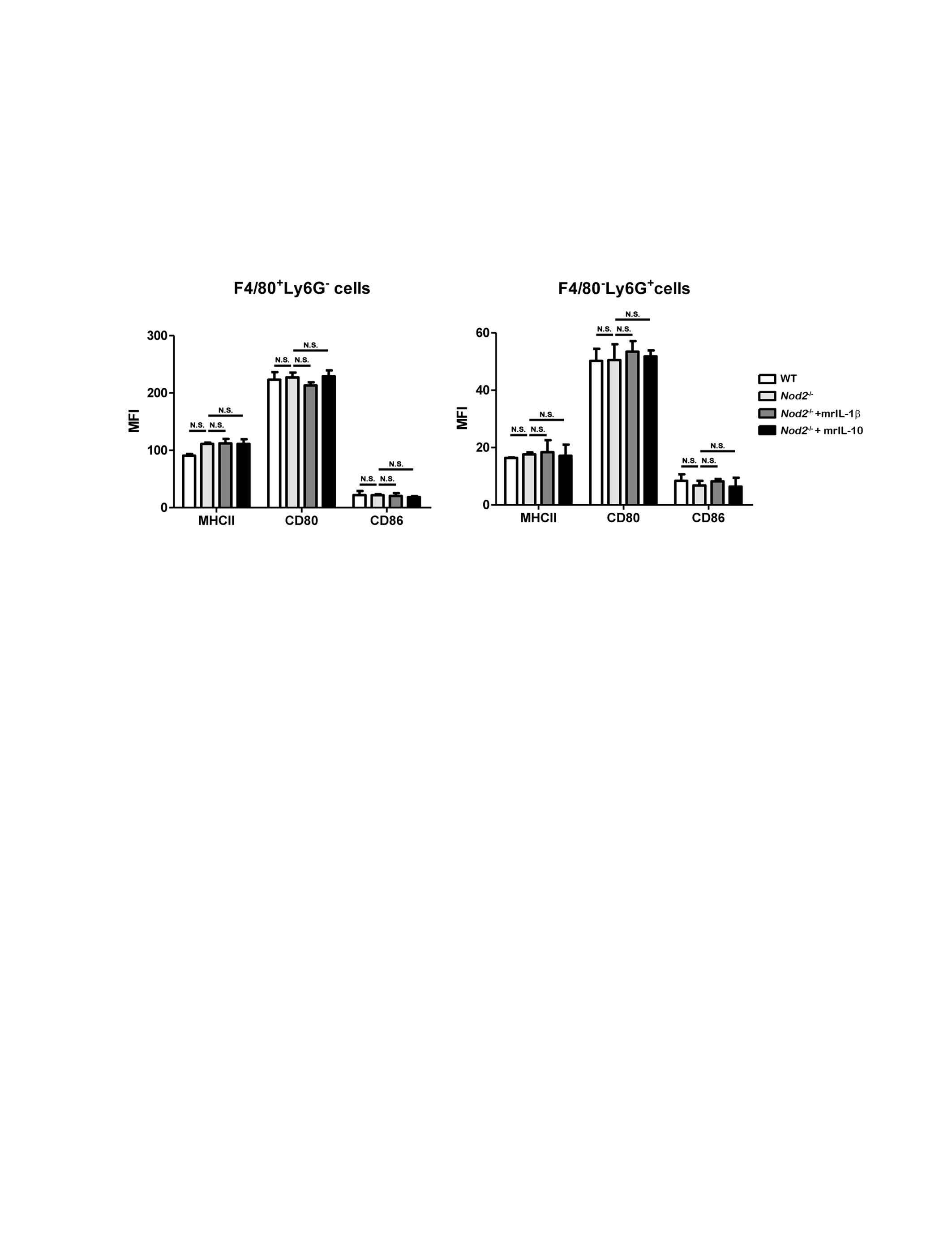

Supplement: Figure S9 — Expression levels of MHC class II, CD80, and CD86 on F4/80+Ly-6G− and F4/80−Ly-6G+ peritoneal cells are similar in WT and nucleotide-binding oligomerization domain (Nod)2−/− mice following cecal ligation and puncture (CLP). Expression levels were estimated on gated F4/80+Ly-6G− and F4/80−Ly-6G+ peritoneal cells obtained from WT B6 or Nod2−/− mice 24 h after CLP. Numbers in diagrams represent mean fluorescence intensity (MFI) for the molecules indicated. (n = 3) Results shown are representative of three independent experiments. N.S.; not significant (one-way ANOVA). (TIF) [file ppat.1003351.s009.tif]
